# Constituents of *Pulicaria inuloides* and Cytotoxic Activities of Two Methoxylated Flavonols

**DOI:** 10.3390/molecules28020480

**Published:** 2023-01-04

**Authors:** Janusz Malarz, Klaudia Michalska, Agnieszka Galanty, Anna Karolina Kiss, Anna Stojakowska

**Affiliations:** 1 Maj Institute of Pharmacology, Polish Academy of Sciences, Smętna Street 12, 31-343 Kraków, Poland; 2 Department of Pharmacognosy, Jagiellonian University Medical College, Medyczna Street 9, 30-688 Kraków, Poland; 3 Department of Pharmacognosy and Molecular Basis of Phytotherapy, Medical University of Warsaw, 1 Banacha Street, 02-097 Warsaw, Poland

**Keywords:** caffeic acid derivatives, caffeoylhexaric acids, caryopyllene derivatives, cytotoxicity, polymethoxylated flavonols, thymol derivatives

## Abstract

Plants of the genus *Pulicaria* are known for providing traditional medicines, spices, herbal teas, and insect deterrents. *Pulicaria inuloides* (Poir.). DC. is one of the less chemically studied species within the genus. Hydroalcoholic extracts from roots and aerial parts of *P. inuloides* were analyzed using the UHPLC-PAD-MS^n^ technique and revealed the presence of six caffeoylquinic and eleven caffeoylhexaric conjugates together with hydroxykaempferol dimethyl ether and quercetagetin trimethyl ether. Moreover, constituents of chloroform extract from the whole *P. inuloides* plants were isolated and identified by spectroscopic methods. One new and four known caryophyllene derivatives, three thymol derivatives, and four polymethoxylated flavonols were found in the analyzed extract. The structure of the new compound was established by spectroscopic methods (HRESIMS, ^1^H NMR, ^13^C NMR, COSY, HSQC, HMBC, NOESY). The cytotoxicity of 6-Hydroxykaempferol 3,7-dimethyl ether and quercetagetin 3,7,3’-trimethyl ether (chrysosplenol C), which are major flavonols isolated from the plant, were tested on prostate epithelial cells (PNT2), prostate cancer cells (DU145 and PC3), human keratinocytes (HaCaT), and melanoma cells (HTB140 and A375). Both flavonols demonstrated moderate cytotoxic activity against PC3 cells (IC_50_ = 59.5 µM and 46.6 µM, respectively). The remaining cell lines were less affected (IC_50_ > 150 µM).

## 1. Introduction

The genus *Pulicaria* Gaertn. (Asteraceae, Inuleae-Inulinae) comprises over 80 species that are native to Europe, Asia, and Africa [1]. Only about one-third of them have been phytochemically investigated so far [2,3,4,5,6,7,8,9,10,11]. Numerous species are endemic to the Mediterranean Basin, and only two: *P. dysenterica* (L.) Bernh. and *P. vulgaris* Gaertn. are widespread in Europe. *P. odora* (L.) Rchb., *P. undulata* (L.) C.A.Mey., *P. incisa* (Lam.) DC. and *P. dysenterica* are a few examples of *Pulicaria* species that have found use in traditional medicine systems, predominantly as anti-inflammatory and antimicrobial agents [3,11,12,13,14]. The phytochemical data on *Pulicaria* mostly concern compositions of essential oils and polyphenolic fractions of plant extracts; the latter often with limited information on structures of the components (measurements of total phenolic and total flavonoid content, tentative identification of the most common compounds). Thymol and caryophyllene derivatives, bisabolene, germacrane, xanthane, pseudoguaiane, and guaiane sesquiterpenoids, as well as diterpenoids and polymethoxylated flavonoids, are the most frequently described secondary metabolites isolated from *Pulicaria* spp. The anti-inflammatory activity of *Pulicaria* extracts is most frequently ascribed to the presence of sesquiterpene lactones and flavonoids [14,15,16,17,18], whereas antimicrobial activity seems to be connected mainly with monoterpenoid constituents [3,12,19,20].

*P. inuloides* (Poir.) DC. is a perennial herb, hemicryptophyte, that is native to North Africa. The flowers of the plant are used in Yemen as a spice [21]. Recently, the evaluation of the leishmanicidal potential of *P. inuloides* has led to the isolation of its active constituents: quercetagetin-3,5,7,3’-tetramethyl ether and 8,9-epoxy-10-isobutyryloxythymyl isobutyrate [22,23]. Data on the chemical composition of the plant are sparse. A study by Galala et al. [24] described the isolation of one kaurane dimer, one derivative of kaurenoic acid in a free and glucosidic form, two methoxylated flavonols, β-sitosterol, and β-sitosterol glucoside. Essential oil from the aerial parts of the plants collected in the Southwest of Algeria has been recently chemically characterized. Δ-Cadinene, α-*epi*-cadinol, and α-cadinol were identified as major constituents of the analyzed oil [25].

Polar extracts from *P. inuloides*, as yet, have been characterized by their total phenolic content, total flavonoid content, antiparasitic, antioxidative, and radical scavenging activity [22,25]. There are no data on the chemical constituents of this part of the plant metabolome. Data on the composition of the non-polar (chloroform) fraction of *P. inuloides* extracts are limited and inconsistent [22,24]. On the other hand, the plant is utilized as a food additive and demonstrates some therapeutic potential. Thus, the objective of the present study was the identification of major polyphenolic metabolites from hydroalcoholic extracts of roots and aerial parts of *P. inuloides*, the isolation of terpenoid and phenolic constituents of chloroform extract from the whole plant, and an examination of the isolated polymethoxylated flavonols in respect to their cytotoxic activity against prostate epithelial and cancer cells, melanoma cell lines, and human keratinocytes.

## 2. Results

### 2.1. Composition of Hydroalcoholic Extracts from P. inuloides

**Figure 1:**
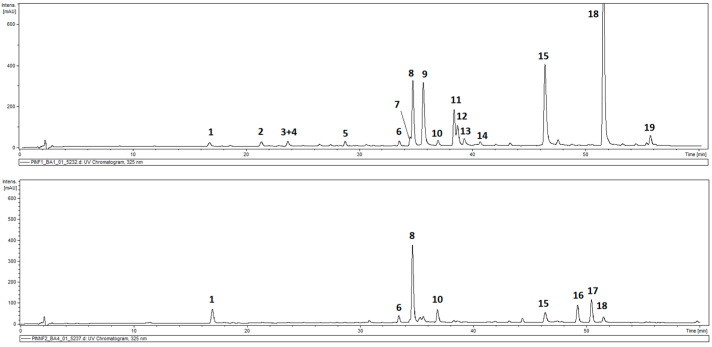
HPLC-UV chromatogram of *Pulicaria inuloides* extracts at a concentration of 10 mg/mL (2 μL injected) acquired at 325 nm: upper part—roots, lower part—aerial parts. Compounds: **1**—5-*O*-caffeoylquinic acid, **2**—dicaffeoylhexaric acid (I), **3**—1,3-di-*O*-caffeoylquinic acid, **4**—dicaffeoylhexaric acid (II), **5**—dicaffeoylhexaric acid (III), **6**—3,4-di-*O*-caffeoylquinic acid, **7**—1,5-di-*O*-caffeoylquinic acid, **8**—3,5-di-*O*-caffeoylquinic acid, **9**—tricaffeoylhexaric acid (I), **10**—4,5-di-*O*-caffeoylquinic acid, **11**—tricaffeoylhexaric acid (II), **12**—tricaffeoylhexaric acid (III), **13**—tricaffeoylhexaric acid (IV), **14**—isobutyryl-dicaffeoylhexaric acid (I), **15**—tetracaffeoylhexaric acid, **16**—hydroxykaempferol dimethyl ether, **17**—quercetagetin trimethyl ether, **18**—isobutyryl-tricaffeoylhexaric acid, and **19**—2-methylbutyryl/isovaleryl-tricaffeoylhexaric acid.

The total phenolic contents in the leaves of *P. inuloides* was estimated as 43.81 ± 2.36 mg GA eq/g DW and was higher than that in the inflorescences (capitula), 29.96 ± 1.0 mg GA eq/g DW. HPLC-DAD-MS^n^ analysis of hydroalcoholic extracts from the roots and aerial parts of the plant revealed the presence of 19 compounds in total, and out of them, 17 demonstrated absorption maxima at 324–328 nm (caffeic acid derivatives). All the detected hydroxycinnamates accumulated in the roots of the plant, and only six were found in the aerial parts. Moreover, two compounds with different UV/Vis spectral properties were observed (Figure 1, Table 1). The two compounds (peaks 16 and 17) that were spotted only in aerial parts of the plant were identified as hydroxykaempferol dimethyl ether (peak 16) and quercetagetin trimethyl ether (peak 17), based on their UV spectra, quasimolecular ions at *m*/*z* 329 [M − H]^−^ and 359 [M − H]^−^ and product ions at *m*/*z* 314 and *m*/*z* 344, respectively. Peak 1 (*m*/*z* = 353 [M − H]^−^) was identified as a signal of 5-*O*-caffeoylquinic acid (5-CQA; IUPAC numbering system), whilst compounds 3, 6–8, and 10 (*m*/*z* = 515 [M − H]^−^), taking into consideration the fragmentation patterns of their quasimolecular ions (Table 1), were recognized as five isomers of di-*O*-caffeoyl quinic acid (DCQA), namely: 1,3-; 3,4-; 1,5-, 3,5-, and 4,5-di-*O*-caffeoylquinic acids [26,27]. Peaks 2, 4, 5, 9, and 11–13 represented compounds that showed a cleavage of two or three caffeoyl [M − H-(2–3 × 162)]^−^ moieties resulting in the *m*/*z* 209 fragments, and were assigned to hexaric acid derivatives: di-*O*-caffeoylhexaric acid I (peak 2), di-*O*-caffeoylhexaric acid II (peak 4), di-*O*-caffeoylhexaric acid III (peak 5), tri-*O*-caffeoylhexaric acid I (peak 9), tri-*O*-caffeoylheharic acid II (peak 11), tri-*O*-caffeoylhexaric acid III (peak 12), and tri-*O*-caffeoylhexaric acid IV (peak 13). Tetra-*O*-caffeoylhexaric acid (peak 15) was identified based on its UV maximum, pseudo-molecular ion mass, and fragmentation pattern identical to those found earlier [28]. The compounds represented by peaks 14 and 18 were isobutyryl-dicaffeoylhexaric acid I and isobutyryl-tricaffeoylhexaric acid judging from the *m*/*z* values of their quasimolecular ions (603 [M − H]^−^ and 765 [M − H]^−^, respectively) and fragmentation ions at *m*/*z* 441, 423, and 279 [28,29]. The compound corresponding to peak 19 demonstrated similar fragmentation patterns to that of 18 except for the fact that the masses of the quasimolecular ion (779 [M − H]^−^) and fragmentation ions at *m*/*z* 617, 455, and 293 were fourteen units higher than those of the analogous ions from isobutyryl-tricaffeoylhexaric acid. Therefore, the compound was tentatively identified as 2-methylbutyryl or 3-methylbutyryl (isovaleryl)-tricaffeoylhexaric acid.

### 2.2. Constituents of a Chloroform Extract from P. inuloides

**Figure 2:**
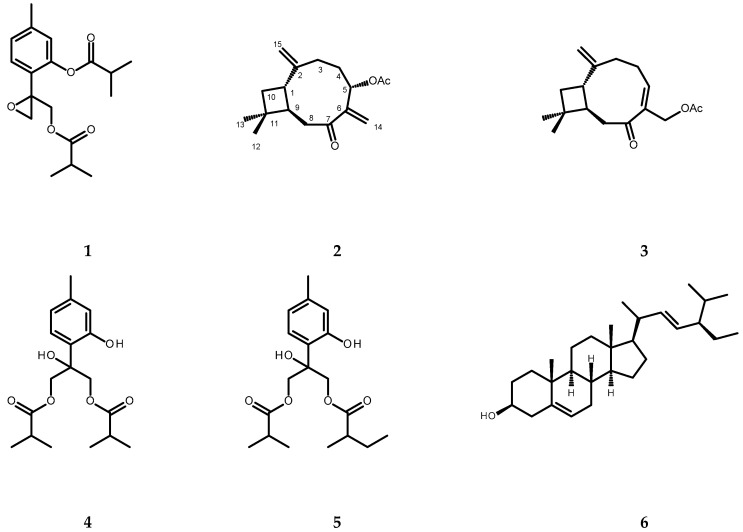

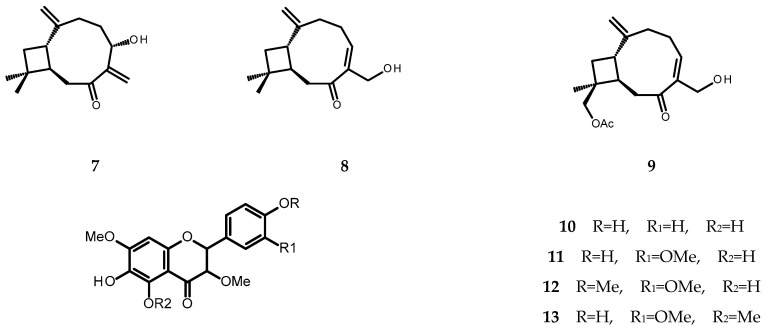
Chemical structures of terpenoid compounds isolated from the chloroform extract of *Pulicaria inuloides*: **1**—8,9-epoxy-10-isobutyryloxythymyl isobutyrate; **2**—(1*S*,9*R*)-5*α*-acetoxycaryophylla-2(15),6(14)-dien-7-one; **3**—(1*S*,9*R*)-14-acetoxycaryophyll-5(6)-en-7-one; **4**—8-hydroxy-9,10-diisobutyryloxythymol; **5**—8-hydroxy-9-isobutyryloxy-10-(2-methylbutyryloxy)thymol; **6**—stigmasterol; **7**—(1*S*,9*R*)-5*α*-hydroxycaryophylla-2(15),6(14)-dien-7-one; **8**—(1*S*,9*R*)-14-hydroxycaryophyll-5(6)-en-7-one; **9**—(1*S*,9*R*)-12*β*-acetoxy-14-hydroxycaryophyll-5(6)-en-7-one; **10**—6-hydroxykaempferol 3,7-dimethyl ether; **11**—quercetagetin-3,7,3′-trimethyl ether (chrysosplenol C); **12**—quercetagetin-3,7,3′,4′-tetramethyl ether; **13**—quercetagetin-3,5,7,3′-tetramethyl ether.

The chromatographic separation of the chloroform extract from whole plants of *P. inuloides* led to the isolation of one new natural product, (1*S*,9*R*)-5*α*-acetoxycaryophylla-2(15),6(14)-dien-7-one (**2**, see Figure 2), and twelve known natural products: 8,9-epoxy-10-isobutyryloxythymyl isobutyrate (**1**), (1*S*,9*R*)-14-acetoxycaryophyll-5(6)-en-7-one (**3**), 8-hydroxy-9,10-diisobutyryloxythymol (**4**), 8-hydroxy-9-isobutyryloxy-10-(2-methylbutyryloxy)thymol (**5**), stigmasterol (**6**), (1*S*,9*R*)-5*α*-hydroxycaryophylla-2(15),6(14)-dien-7-one (**7**), (1*S*,9*R*)-14-hydroxycaryophyll-5(6)-en-7-one (**8**), (1*S*,9*R*)-12*β*-acetoxy-14-hydroxycaryophyll-5(6)-en-7-one (**9**), 6-hydroxykaempferol 3,7-dimethyl ether (**10**), quercetagetin 3,7,3′-trimethyl ether (**11**, chrysosplenol C), quercetagetin 3,7,3′4′-tetramethyl ether (**12**), and quercetagetin 3,5,7,3′-tetramethyl ether (**13)** (for structures see Figure 2). The structure of compound **2** was established based on the analysis of its spectral data (1D- and 2D-NMR, specific rotation measurement). The remaining compounds were identified by the direct comparison of their spectral data (^1^H NMR, specific rotation, UV spectra) to those found in the literature [7,30,31,32,33,34,35,36,37,38,39,40].

#### Structure Elucidation

Compound **2** was isolated as a colorless amorphous solid. An adduct ion peak at *m*/*z* 299.1620 [M + Na]^+^, which was observed in the HRESIMS spectrum of **2,** corresponded to the molecular formula of C_17_H_24_O_3_Na (calculated mass 299.1623). The molecular formula of the compound, established as C_17_H_24_O_3_ (Figure 2), indicated six degrees of unsaturation that might be accounted for the two rings system, two olefinic double bonds, and two carbonyl groups.

The ^13^C NMR spectrum (Table 2), together with HSQC data, revealed the presence of seventeen carbon atoms assigned to three methyl groups (δ_C_ 21.16, 23.36, 29.95), six methylenes (δ_C_ 30.24, 33.83, 38.46, 41.72, 111.82, 120.73), three methines, including one esterified group (δ_C_ 40.10, 51.57, 72.20), three quaternary carbons (δ_C_ 33.55, 150.34, 152.95), and two carbonyls (δ_C_ 169.58 and 204.70). The signals at δ_C_ 111.82, 120.73, 150.34, and 152.95 indicated the presence of two double bonds. The HMBC spectrum confirmed the location of the quaternary carbons at C-2, C-6, and C-11 based on the correlations from H-1*β*, H-3*α*, H-3*β*, H-4*α*, H-10’, and H-10” to C-2 as well as from H-4*β*, H-8*α*, H-14a, and H-14b to C-6; H-8*α*, H-8*β*, H-10’, and H_3_-13 to C-11. The HMBC correlations from H-5*β*, H-8*α*, H-8*β*, H-14a, and H-14b to the carbon atom at δ_C_ 204.7 supported the location of the carbonyl group at C-7. The signal of the second carbonyl (δ_C_ 169.58) correlated with the protons of the acetoxy methyl group at δ_H_ 2.12 (Table 2; Figures S8 and S9 Supplementary Materials).

**Figure 3:**
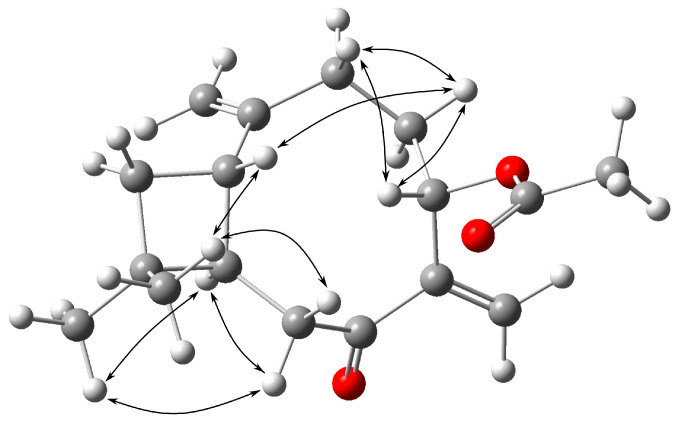
Key NOESY correlations for **2**.

The NOESY spectrum verified the proximity of H-1*β* to H-4*β* and H-12*β*; H-12*β* to H-1*β* and H-8*β*; H-4*β* to H-1*β*, H-3*β*, and H-5*β*; H-3*β* to H-4*β* and H-5*β*, as well as the proximity of H-9*α* to H-8*α* and H-13*α* (Figure 3).

On the basis of the above data, compound **2** was deduced to be a new caryophyllene derivative, (1*S*,9*R*)-5*α*-acetoxycaryophylla-2(15),6(14)-dien-7-one.

### 2.3. Cytotoxic Activities of 6-Hydroxykaempferol 3,7-Dimethyl Ether and Quercetagetin 3,7,3’-Trimethyl Ether

Compounds **10** and **11**, as major flavonoid constituents of the plant, were assayed for their cytotoxic effects against normal and cancer cells (Table 3). Normal prostate epithelial cells (PNT-2), two prostate cancer cell lines (DU145 and PC3), human keratinicytes (HaCaT), and two melanoma cell lines were used in the experiments. The tested compounds were not active toward the keratinocytes and melanoma cells at a dose range of 5–100 μg/mL. Both **10** and **11** demonstrated moderate cytotoxic activity against PC3 cells (IC_50_ = 19.64 ± 0.83 and 16.79 ± 0.77 μg/mL, respectively). However, normal prostate epithelial cells and DU145 cells were less susceptible.

## 3. Discussion

Recent phytochemical investigations of *P. inuloides* have led to the identification of components in the essential oils from aerial parts of the plant [25] and to the isolation of several polyphenolic and terpenoid constituents, including *ent*-kaurane diterpenoids, *β*-sitosterol, daucosterol, and methoxylated flavonols (**10**,**11**,**13**) [22,24]. The bioguided fractionation of the *P. inuloides* root extract yielded 8,9-epoxy-10-isobutyryloxythymyl isobutyrate (**1**) as a constituent responsible for the antileishmanial activity of the plant [23]. Despite the interest in the biological activity of *P. inuloides* extracts, data on secondary metabolites produced by the plant are sparse.

Plants of the Inuleae tribe are rich in hydroxycinnamates that are largely responsible for the antioxidant activity of the plant extracts, and possess potential health-enhancing properties [28,29,41,42,43]. To our knowledge, hydroxycinnamates of *P. inuloides* have not been studied before. Extracts from *P. dysenterica*, analyzed using high-performance liquid chromatography coupled with electrospray ionization and time-of-flight mass spectrometry (HPLC-ESI-TOF-MS), revealed the presence of three isomers of chlorogenic acid (CQA) and four dicaffeoylquinic acids (DCQAs) [11]. However, structural details (such as positions of substitution) were not given. Another metabolomic study, devoted to the composition of extracts from *P. crispa* and *P. incisa*, enabled the tentative identification of two isomeric CQAs, caffeic acid, caffeoyl-*O*-shikimic acid, *O*-coumaroylquinic acid, dehydro-*O*-dicaffeoyl-hydroxyferulic acid, tri-*O*-caffeoyl-hydroxyferulic acid, five DCQA isomers, three isomers of *p*-coumaroyl-*O*-caffeoylquinic acid, CQA methyl ether, and two tri-*O*-caffeoylquinic acids [9]. Again, more detailed structural data are missing.

A reducing capacity (TPC) of *P. inuloides* dry leaves, assessed as 43.81 ± 2.36 mg GA eq/g, was comparable to that of chicory leaves or young lettuce leaves [44,45] that are rich in caffeic acid derivatives. Hydroalcoholic extracts from the roots and aerial parts of *P. inuloides*, analyzed in the present study, contained chlorogenic acid (5-CQA) as a sole isomer of CQA, DCQAs, di- and tricaffeoylhexaric acids (DCHAs and TCHAs), and their derivatives. The aerial parts of the plant contained: 5-CQA, 3,4-DCQA, 3,5-DCQA, 4,5-DCQA, tetracaffeoylhexaric acid, and one acylated TCHA, whereas the roots additionally accumulated 1,3- and 1,5-DCQA, three isomeric DCHAs, TCHAs (four isomers), and another acylated TCHA. The caffeoylhexaric acids and their acylated deivatives seem to be typical constituents of the Inuleae plants [28,29,43].

Except for hydroxycinnamates, HPLC-DAD-MS^n^ analysis of the extract from aerial parts of *P. inuloides* showed the presesnce of two flavonoids: hydroxykaempferol dimethyl ether and quercetagetin trimethyl ether that correspond to 6-hydroxykaempferol 3,7-dimethyl ether (**10**) and quercetagetin 3,7,3’-trimethyl eter (**11**) isolated from the CHCl_3_ extract of the plant. These two compounds were also isolated by Galala et al. [24] from aerial parts of *P. inuloides,* and there seemed to be major flavonoid constituents in the plant. According to a study on lipophilic and vacuolar flavonoids from the European *Pulicaria* species [46], 6-hydroxykaempferol 3,7-dimethyl ether is a constituent of *P. dysenterica*, and chrysosplenol C (**11**) is the most common flavonoid in European *Pulicaria* plants. The latter compound was the subject of several studies that revealed its antiviral [47], cytotoxic [48,49], and inotropic [50] activity. Quercetagetin-3,5,7,3’-tetramethyl ether (**13**) was isolated from aerial parts of *P. inuloides* as a compound responsible for the antileishmanial activity of the examined extract [22]. Its isomer, quercetagetin-3,7,3’,4’-tetramethyl ether (**12**), was isolated from *P. inuloides* for the first time, although the compound was detected earlier in some other species of *Pulicaria* [46].

Methoxylated flavonols of *Artemisia annua* L. have recently raised some interest as compounds active against different cancer cell lines in vitro [51,52]. The inhibition of topoisomerase IIα and ERK1/2-mediated apoptosis have been proposed as a possible explanation for their activity. This prompted us to investigate the cytotoxic activity of **10** and **11** against normal and cancer cell lines of prostate and skin origin. The results (Table 3) indicated some selectivity of the cytotoxic effect exerted by the tested compounds. The selective activity of chrysosplenol C (**11**) against PC3 cells is worth further studies.

Caryophyllene derivatives, although known as constituents of *Pulicaria* spp., have not been previously found in *P. inuloides*. Examined in this study, a chloroform extract from whole *P. inuloides* yielded five compounds of this structural type, including the new one, (1*S*,9*R*)-5*α*-acetoxycaryophylla-2(15),6(14)-dien-7-one (**2**). (1*S*,9*R*)-14-Acetoxycaryophyll-5(6)-en-7-one (**3**) has been described as a metabolite of *P. scabra* [31], *P. arabica* [32], and *P. dysenterica* [33]. Zardi-Bergaoui et al. [7] from fresh aerial parts of *P. vulgaris* isolated (1*S*,9*R*)-5*β*-hydroxycaryophylla-2(15),6(14)-dien-7-one as a new compound named pulicaryenne A. The ^1^H NMR spectrum of the compound was identical to that of **7**. However, measurements of specific rotation confirmed the *β*-orientation of the proton at C-5 of **2** and suggested that compound **7** should be identified as (1*S*,9*R*)-5*α*-Hydroxycaryophylla-2(15),6(14)-dien-7-one. (1*S*,9*R*)-14-Hydroxycaryophyll-5(6)-en-7-one (**8**) is a known compound isolated previously from *P. vulgaris* Gaertn. [7], *P. scabra* [31], and *P. arabica* [32]. *P. vulgaris*, *P. arabica*, *P. dysenterica,* and *P. paludosa* accumulated in **9** as one of their metabolites [7,32,33,53]. Compounds **8** and **9** demonstrated moderate cytotoxic activity against HeLa and A549 cells in vitro and weak anticholinesterase activity [7].

Thymol derivatives are commonly distributed in plants of the Inuleae tribe, including *Pulicaria* spp. [2,3,6,28,36]. 8,9-Epoxy-10-isobutyryloxythymyl isobutyrate (**1**) is one of the most frequently isolated compounds of this type. Apart from the already mentioned antileishmanial [23] and moderate antimicrobial [54] effects, the compound demonstrated promising activity as an inhibitor of pathological activities of protein kinase B (Akt) and extracellular signal-regulated kinases (ERKs) in human melanoma cells [55]. 8-Hydroxy-9,10-diisobutyryloxythymol (**4**), which may be a product of the degradation of the corresponding epoxide (**1**) [55], possess moderate antimicrobial and anti-inflammatory activity [34,56,57]. Moreover, **4** showed some therapeutical potential as an inhibitor of the p53 tumor suppressor inactivation [58].

In summary, *P. inuloides* is a plant rich in phenolic and terpenoid metabolites that demonstrates a wide range of biological activities. The most important seems to be the modulatory effect on subcellular signaling pathways that may hold promise for future therapeutic use. 

## 4. Materials and Methods

### 4.1. General Methods 

NMR spectra were recorded either in CDCl_3_ or in DMSO-d_6_ on a Bruker AVANCE III HD 400 (resonance frequency 400.17 MHz for ^1^H). Optical rotation was determined using a PolAAr31 polarimeter (Optical Activity Ltd., Ramsey, UK). RP-HPLC separations were performed using an Agilent 1200 Series HPLC system (Agilent Technologies, Santa Clara, CA, USA) equipped with a diode array detector (PAD). Analytical chromatographic separations were carried out either on a Kinetex XB-C18 column (4.6 × 250 mm, 5 μm total particle size; Phenomenex, Torrance, CA, USA; nonpolar compounds) or on a Zorbax Eclipse XDB-C18 column (4.6 × 150 mm; Agilent Technologies, USA; phenolic compounds). Semipreparative RP-HPLC was conducted on a Vertex Plus column (Eurospher II 100-5 C18, 8 × 250 mm; Knauer GmbH, Berlin, Germany), with an isocratic elution, using MeOH-H_2_O mixtures of different polarities, at a flow rate of 1.0–2.0 mL min^−1^. The column was coupled to a Knauer P4.1S pump and a dual wavelength UV/VIS detector operating at 210 and 260 nm. Conventional column chromatography was carried out on Silica gel 60 (0.063–0.2 mm, Merck, Germany) and Sephadex LH-20 (GE Healthcare, Uppsala, Sweden). TLC separations were performed using precoated plates (Silica gel 60 without fluorescence indicator, Art. No 5553, Merck, Germany).

### 4.2. Chemicals and Solvents

Chlorogenic acid (5-O-CQA, purity > 97% by HPLC), caffeic acid (purity ≥ 95%), and 1,3-DCQA (cynarin, purity > 99% by HPLC) were purchased from Roth (Karlsruhe, Germany). The Folin–Ciocalteu reagent was supplied by Sigma-Aldrich Co. (St. Louis, MO, USA). MeOH, EtOAc, and CHCl_3_ of an analytical grade were purchased from Avantor Performance Materials S.A. (Gliwice, Poland). Water was purified by a Mili-Q system (Milipore Corp., Bedford, MA, USA). Hexane of the analytical grade, MeOH, and MeCN of the HPLC grade, as well as formic acid and glacial acetic acid were bought from Merck (Darmstadt, Germany). 

### 4.3. Plant Material

The seeds of *Pulicaria inuloides* DC. were delivered by the Jerusalem Botanical Gardens of the Hebrew University of Jerusalem (Jerusalem, Israel). Plants obtained from the seeds were cultivated in the greenhouse of the Garden of Medicinal Plants, Maj Institute of Pharmacology, Polish Academy of Sciences in Kraków (Cracow, Poland), and the voucher specimens (3/2019, 10/2020) were deposited there. Plants (roots and aerial parts) were collected at blooming in July 2021 and dried under shade at room temperature.

### 4.4. Estimation of Total Phenolic Content (TPC)

The total phenolic content (the reducing capacity of the plant material) was estimated using a Folin–Ciocalteu colorimetric method. The dry plant material (0.01 g) was extracted twice for 2 h, with 2 mL of 80% MeOH containing 1% HCl, at room temperature, on a reciprocal shaker. The combined extracts were further analyzed as described by Velioglu et al. [59]. In brief, a 0.1 mL aliquot of the extract was mixed with 0.75 mL of the Folin–Ciocalteu reagent diluted with water (10-fold). After 5 min, 0.75 mL of the sodium bicarbonate solution (60 g/L) was added to the mixture. After the following two hours at room temperature, absorbance was measured at 725 nm. The results are expressed as the mg of gallic acid equivalents (GAeq) per 1 g of the plant material dry weight and are the means of three measurements (±SD).

### 4.5. Phenolic Compounds Analysis by HPLC 

#### 4.5.1. Preparation of Samples for HPLC-PAD and UHPLC-PAD-MS^n^ Analysis

The dry and pulverized plant material (0.1 g) was extracted twice with 10 mL of 70% MeOH at room temperature for 3 h on a rotary shaker (100 r.p.m.). The extracts were combined and evaporated to dryness under a reduced pressure to give a residue that was either redissolved in 1 mL of 70% MeOH and centrifuged (11.340× *g*, 5 min) prior to analytical HPLC-PAD separation or its aliquote (0.01 g) was dissolved in a mixture of MeOH and 0.1% HCOOH (8:2), before being filtered through a 0.45 μm Chromafil membrane (Machery-Nagel, Duren, Germany) and subjected to UHPLC-PAD-MS^n^ analysis.

#### 4.5.2. Characterization of *P. inuloides* Shoot and Root Extracts by HPLC-DAD-MS^n^ Method

UHPLC-PAD-MS^n^ analysis was performed on a UHPLC-3000 RS system (Dionex, Germany) with PAD detection and an AmaZon SL ion trap mass spectrometer with ESI interface (Bruker Daltonik GmbH, Bremen, Germany). Separation was performed on a Zorbax SB-C18 column (150 × 2.1 mm, 1.9 μm) Agilent (USA). The column temperature was 25 °C. The mobile phase (A) was water /formic acid (100:0.1, *v*/*v*), and the mobile phase (B) was acetonitrile/formic acid (100:0.1, *v*/*v*). A gradient system was used: 0–60 min, 5–40% B. The flow rate was 0.2 mL/min. The column was equilibrated for 7 min between injections. UV spectra were recorded over a range of 200–450 nm, and chromatograms were acquired at 325 nm. The LC eluate was introduced directly into the ESI interface without splitting. The nebuliser pressure was 40 psi; the dry gas flow was 9 L/min; the dry temperature was 300 °C; and the capillary voltage was 4.5 kV. Analysis was carried out using a scan from *m*/*z* 90 to 2200. Compounds were analyzed in the negative ion mode. The MS^2^ fragmentation was obtained for the most abundant ion at the time. 

### 4.6. Isolation of Chemical Constituents from a Chloroform Extract of P. inuloides

Dried and pulverized whole plants of *P. inuloides* (118.4 g) were extracted five times with 0.7 L of CHCl_3_ at room temperature with shaking. The combined extracts were concentrated in vacuo at 40 °C, providing c. 4.6 g of an oily residue. The residue was subjected to CC on silica using gradients of EtOAc in n-hexane (up to 100% EtOAc) and subsequently MeOH in EtOAc (up to 10% of MeOH). The separated fractions (50 mL each) were combined, as shown by TLC and further fractionated if required. Fractions 46–49, eluted with n-hexane-EtOAc 97:3 (*v*/*v*), were subjected to a semipreparative RP-HPLC (solvent system: MeOH-H_2_O 7:3; *v*/*v*; isocratic elution, flow rate: 2 mL/min) to give subfractions containing monoterpene thymol derivatives **1** (t*_R_* = 47.5 min; 6.8 mg) and **4** (t*_R_* = 24.5 min; 6.1 mg) together with a new caryophyllene derivative **2** (t*_R_* = 62.5 min; 1.5 mg). Fractions 54–55, eluted from the silica gel column with n-hexane-EtOAc 19:1 (*v*/*v*), after HPLC separation, conducted as described above, yielded a mixture of compounds containing **3** as a major constituent (t*_R_* = 12.0 min; 4.6 mg). The mixture was not further separated as the signals of the compound were clearly visible in the corresponding ^1^H NMR spectrum. Combined fractions 56–59 (n-hexane-EtOAc 19:1; *v*/*v*) were partly dissolved in MeOH and the MeOH insoluble residue was identified as stigmasterol (**6**, 25.9 mg). From the fractions 64 to 71, eluted with the same solvent as 54–59 (n-hexane-EtOAc 19:1; *v*/*v*), additional amounts of **4** (t*_R_* = 28.0 min; 1.6 mg) along with **5** (t*_R_* = 39.0 min; 1.4 mg) were obtained after semipreparative HPLC separation (MeOH-H_2_O 7:3, *v*/*v*; 1.8 mL/min). Fractions 75–77, eluted with n-hexane-EtOAc 9:1 (*v*/*v*), subjected to the same HPLC separation procedure as fractions 64–71, gave **4** (7.6 mg), **5** (1.8 mg) and **7** (t*_R_* = 32 min; 1.9 mg). Compound **8** (t*_R_* = 67.5 min; 7.9 mg) was isolated from the fractions 82 to 85 (n-hexane-EtOAc 9:1; *v*/*v*) after purification by semipreparative HPLC (MeOH-H_2_O 3:2, *v*/*v*; 1.7 mL/min). Fractions eluted with n-hexane-EtOAc 4:1 (*v*/*v*) were subjected to semipreparative HPLC (MeOH-H_2_O 3:2, *v*/*v*; 1.5 mL/min) to yield **9** (t*_R_* = 34.0 min; 5.4 mg). 

The further elution of the silica gel column allowed the isolation of methoxylated flavonols. Compound **10** (t*_R_* = 40.0 min; 17.9 mg) was obtained after the preparative separation (MeOH-H_2_O 3:2, *v*/*v*; 1.0 mL/min) of fractions 138–142 (eluted with n-hexane-EtOAc 7:3; *v*/*v*). Fractions 143–169, eluted with the same solvent, were subjected to semipreparative HPLC (MeOH-H_2_O 3:2, *v*/*v*; 1.5 mL/min) to give **11** (t*_R_* = 27.5 min; 35.6 mg) **12** (t*_R_* = 44 min; 4.5 mg) and an additional amount of **10** (33.0 min; 2.9 mg). Fractions 186–201 (88 mg), eluted from the silica gel column with n-hexane-EtOAc 1:1 (*v*/*v*), were further separated by CC on Sephadex LH-20 using a gradient of MeOH in H_2_O (from 50% up to 75% of MeOH). Subfractions S3-S7 were eluted with 50% MeOH and yielded pure **13** (12 mg). 

#### Characterization of the Isolated Caryophyllene Derivatives

(1*S*,9*R*)-5*α*-Acetoxycaryophylla-2(15),6(14)-dien-7-one (**2**). Colorless, amorphous solid: [α]_D_^24^.^8^: −100.7° (c = 0.33, CHCl_3_); UV (MeCN-H_2_O) λ_max_ 218 nm, 317 nm; ^1^H- and ^13^C-NMR: Table 2; 1D and 2D NMR: Supplementary Materials Figures S2–S9; HRESIMS (pos. mode) *m*/*z* 299.1620 [C_17_H_24_O_3_Na]^+^, calc. 299.1623, Supplementary Materials Figure S1.

(1*S*,9*R*)-5*α*-Hydroxycaryophylla-2(15),6(14)-dien-7-one (**7**). Colorless, amorphous solid: [α]_D_^25^: −41.3° (c = 0.47, CHCl_3_); UV (MeCN-H_2_O) *λ*_max_ 314 nm; ^1^H NMR: Supplementary Materials Figure S17.

(1*S*,9*R*)-14-Hydroxycaryophyll-5(6)-en-7-one (**8**). Colorless, amorphous solid: [α]_D_^25^.^2^: −128.8° (c = 2.13, CHCl_3_); UV (MeCN-H_2_O) *λ*_max_ 232 nm, 313 nm; ^1^H NMR: Supplementary Materials Figure S18.

(1*S*,9*R*)-12*β*-acetoxy-14-hydroxycaryophyll-5(6)-en-7-one (**9**). Colorless, amorphous solid: [α]_D_^25^.^4^: −203.1° (c = 1.50, CHCl_3_); UV (MeCN-H_2_O) *λ*_max_ 233 nm, 310 nm, 327 nm; 1D and 2D NMR: Supplementary Materials Figures S10–S15.

### 4.7. Cell Culture and Cytotoxicity Assessment

Cytotoxic activity was tested on human cancer and normal cells, namely: prostate cancer cell lines DU145 (ATCC HTB-81) and PC3 (ATCC CRL-1435), prostate epithelial cells PNT-2 (ECACC 95012613), melanoma cell lines A375 (ATCC CRL-1619) and HTB140 (ATCC Hs 294T) and skin keratinocytes HaCaT (obtained as a kind gift from prof. Marta Michalik, Department of Cell Biology, Jagiellonian University). DU145 cells were grown in a modified Eagle’s medium with a low (1.0 g/L) glucose concentration, as well as PC3 and PNT-2 cells in Dulbecco’s modified Eagle’s media: F12 HAM nutrient mixture. At the same time, melanoma cells and keratinocytes were maintained in a modified Eagle’s medium with a high (4.5 g/L) glucose concentration. The culture media (all supplied by Sigma-Aldrich Co.; St. Louis, MO, USA) contained antibiotics and 10% fetal bovine serum (FBS). All cultures were maintained at 37 °C in a humidified 5% CO_2_-containing atmosphere.

The examined flavonols were diluted in the culture media from freshly made stock solutions in MeOH (10 mg/mL) to the working concentrations (from 5 to 100 μg/mL).

Cells were seeded in 96-well plates (1.5 × 10^4^ cells/well) and preincubated for 24 h (37 °C, 5% CO_2_). Then, the culture medium was replaced with a fresh medium containing different concentrations of the tested compounds (5–100 µg/mL). The incubation lasted 48 h. Cell viability was measured by an MTT assay, as previously described [60]. The absorbance at 490 nm was measured using a Synergy II Biotek (BioTek Instruments, Winooski, VT, USA) microplate reader. Cytotoxic activity was assessed based on the cell viability expressed as the percentage of living cells. The results were the means of three independent measurements (±SD). Doxorubicin (Ebewe Pharma GmbH., Unterach, Austria) was used as a reference cytostatic drug. The IC_50_ values were determined by plotting the percentage viability of the cells versus the concentration and adequate calculations made using either Excel or the AAT Bioquest website program (https://www.aatbio.com/tools/ic50-calculator, accessed on 4 September 2022).

## Figures and Tables

**Table 1:** Retention times, UV maxima and MS^n^ data (in the negative ion mode) for the compounds present in *Pulicaria inuloides* extracts.

	Compound	t_R_ [min]	UV [nm]	[M − H]^−^	Product Ion Main Peaks ^1^	R ^2^	A ^3^
1	5-*O*-caffeoylquinic acid (5-CQA)	16.8	325	353	**191**	**+**	**+**
2	dicaffeoylhexaric acid (I)	21.4	323	533	353, **371**, 209, 191	**+**	**−**
3	1,3-di-*O*-caffeoylquinic acid (1,3-DCQA)	23.8	323	515	**353**, 335, 191, 179	**+**	**−**
4	dicaffeoylhexaric acid (II)	23.8	327	533	353, **371**, 209, 191	**+**	**−**
5	dicaffeoylhexaric acid (III)	28.9	327	533	353, **371**, 209, 191	**+**	**−**
6	3,4-di-*O*-caffeoylquinic acid (3,4-DCQA	33.4	326	515	**353**, 335, 299, 255, 203, 173	**+**	**+**
7	1,5-di-*O*-caffeolyquinic acid (1,5-DCQA)	34.6	328	515	**353**, 335, 191	**+**	**−**
8	3,5-di-*O*-caffeoylquinic acid (3,5-DCQA)	34.7	327	515	**353**, 191, 179	**+**	**+**
9	tricaffeoylhexaric acid (I)	35.6	327	695	**533**, **371**, 209	**+**	**-**
10	4,5-di-*O*-caffeoylquinic acid (4,5-DCQA)	37.0	327	515	**353**, 317, 299, 255, 203, 191, 179,173	**+**	**+**
11	tricaffeoylhexaric acid (II)	38.5	328	695	**533**, **371**, 353, 209	**+**	**−**
12	tricaffeoylhexaric acid (III)	38.9	326	695	**533**, **371**, 353, 209	**+**	**−**
13	tricaffeoylhexaric acid (IV)	39.4	328	695	**533**, **371**, 353, 209	**+**	**−**
14	isobutyryl-dicaffeoylhexaric acid (I)	40.7	328	603	**441**, 423, 353, 335, 279, 191	**+**	**−**
15	tetraceffeoylhexaric acid	46.4	328	857	**695**, 533, 371	**+**	**+**
16	hydroxykaempferol dimethylether	49.4	340	329	**314**	**−**	**+**
17	quercetagetin trimethylether	50.6	350	359	**344**	**−**	**+**
18	isobutyryl-tricaffeoylhexaric acid	51.6	328	765	**603**, **441**, 423, 353, 279	**+**	**+**
19	2-methylbutyryl/isovaleryl-tricaffeoylhexaric acid	55.6	328	779	**617**, **455**,353, 293, 191	**+**	**−**

^1^ Ions in bold—most abundant ion peak; ^2^ roots; ^3^ aerial parts; **+** detected in the extract; **−** not detected in the extract.

**Table 2:** ^1^H NMR (400.17 MHz) and ^13^C NMR (100.63 MHz) data of compound **2** in CDCl_3_.

Position	δ_H_ (ppm), *J* (Hz)	δ_C_ (ppm)	HMBC (H → C)
1	2.58 m	40.10	C-2, C-15
2	-	152.95	-
3α	1.92 m	30.24	C-2
3β	2.20 m		C-2, C-15
4β	1.78 m	33.83	C-5, C-6
4α	1.98 ^a^ m		C-2
5β	5.77 m	72.20	C-7
6	-	150.34	-
7	-	204.70	-
8α	2.51 dd (11.2, 5.2)	41.72	C-1, C-6, C-7, C-9, C-11
8β	3.00 dd (11.2, 11.2)		C-1, C-7, C-9, C-11
9	1.95 ^a^ m	51.57	C-6
10′	1.82 ^b^ m	38.46	C-1, C-2, C-9, C-11, C-12, C-13
10″	1.84 ^b^ m		C-2, C-9, C-12, C-13
11	-	33.55	-
12	1.09 s	23.36	C-9, C-13
13	1.08 s	29.95	C-10, C-11, C-12
14a	5.74 d (1.2)	120.73	C-5, C-6, C-7
14b	5.90 brs		C-5, C-6, C-7
15a	4.82 brs	111.82	C-1, C-3
15b	4.85 brs		C-1, C-3
OAc_CO	-	169.58	-
OAc_CH_3_	2.12 s	21.16	C- OAc_CO

^a,b^ Signals overlapped.

**Table 3:** Cytotoxicities of **10** and **11** against human normal and cancer cell lines after the 48 h treatment (5–100 μg/mL).

Compound	IC_50_ (μg/mL)					
	Prostate Normal and Cancer Cells			Keratinocytes and Melanoma Cells		
	PNT-2	DU145	PC3	HaCaT	A375	HTB140
**10**	74.62 ± 1.87	83.62 ± 1.02	19.64 ± 0.83 (59.51) *	>100	>100	>100
**11**	82.38 ± 2.42	54.87 ± 0.23	16.79 ± 0.77 (46.46) *	>100	>100	>100
**Doxorubicin**	1.38	3.18	>50	4.68	0.59	5.71

* IC_50_ (µM).

## Data Availability

The raw data that support the findings of this study are available from the authors (A.G., J.M., A.S.), upon reasonable request.

## Supplementary Materials

The following are available online at https://www.mdpi.com/article/10.3390/molecules28020480/s1, Figures S1–S27 showing HRESIMS of **2** and NMR spectra of **1**–**13**.

## Sample Availability

Samples of the compounds are not available.
