# Supplementary material for: Constituents of Pulicaria inuloides and Cytotoxic Activities of Two Methoxylated Flavonols
Source: Molecules. 2023 Jan 4;28(2):480. doi: 10.3390/molecules28020480 (PMC9866601; doi:10.3390/molecules28020480)
Supplement: Supplementary file 1 [file molecules-28-00480-s001.zip › molecules-2102373-supplementary.pdf]

Constituents of *Pulicaria inuloides* and cytotoxic activities of two methoxylated flavonols

Janusz Malarz, Klaudia Michalska, Agnieszka Galanty, Anna K. Kiss and Anna Stojakowska

SUPPLEMENTARY MATERIAL

Figure S1. LRESIMS and HRESIMS of compound **2**.

Figure S2. ^{1}H NMR spectrum of compound **2** in $CDCl_3$.

Figure S3. ^{13}C NMR spectrum of compound **2** in $CDCl_3$.

Figure S4. ^{1}H-^{1}H COSY spectrum of compound **2**.

Figure S5. COSY correlations for compound **2**.

Figure S6. NOESY spectrum of compound **2**.

Figure S7. HSQC spectrum of compound **2**.

Figure S8. HMBC spectrum of compound **2**.

Figure S9. HMBC correlations for compound **2**.

Figure S10. ^{1}H NMR spectrum of compound **9** in $CDCl_3$.

Figure S11. ^{13}C NMR spectrum of compound **9** in $CDCl_3$.

Figure S12. ^{1}H-^{1}H COSY spectrum of compound **9**.

Figure S13. NOESY spectrum of compound **9**.

Figure S14. HSQC spectrum of compound **9**.

Figure S15. HMBC spectrum of compound **9**.

Figure S16. ^{1}H NMR spectrum of the mixture containing compound **3** in $CDCl_3$.

Figure S17. ^{1}H NMR spectrum of compound **7** in $CDCl_3$.

Figure S18. ^{1}H NMR spectrum of compound **8** in $CDCl_3$.

Figure S19. ^{1}H NMR spectrum of compound **1** in $CDCl_3$.

Figure S20. ^{1}H NMR spectrum of compound **4** in $CDCl_3$.

Figure S21. ^{1}H NMR spectrum of compound **5** in $CDCl_3$.

Figure S22. ^{1}H NMR spectrum of compound **6** in $CDCl_3$.

Figure S23. ^{1}H NMR spectrum of compound **10** in $CDCl_3$.

Figure S24. ^{1}H NMR spectrum of compound **11** in $CDCl_3$.

Figure S25. ^{1}H NMR spectrum of compound **12** in $CDCl_3$.

Figure S26. ^{1}H NMR spectrum of compound **13** in $CDCl_3$.

Figure S27. ^{1}H NMR spectrum of compound **13** in DMSO-d_6.

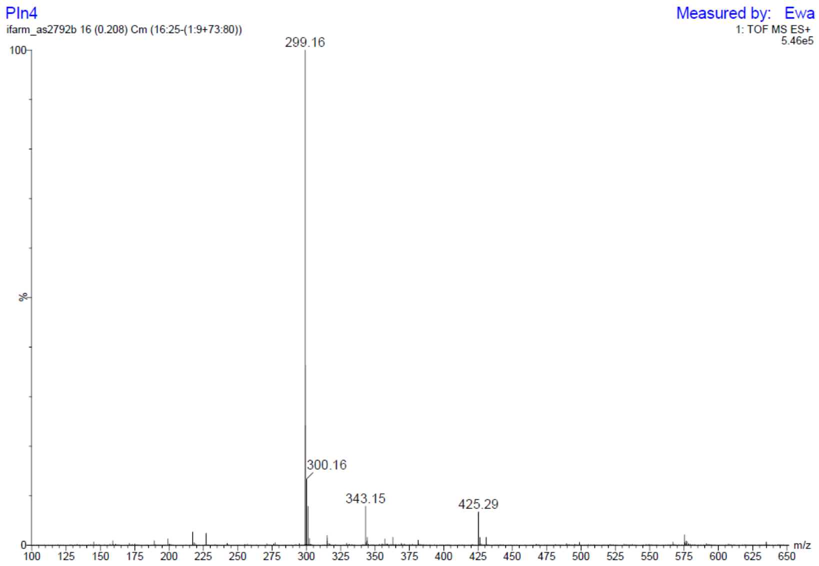

Single Mass Analysis

Tolerance = 3.0 PPM / DBE: min = -1.5, max = 50.0

Element prediction: Off

Number of isotope peaks used for i-FIT = 3

Monoisotopic Mass, Even Electron Ions

48 formula(e) evaluated with 1 results within limits (up to 50 best isotopic matches for each mass)

Elements Used:

C: 0-200 H: 0-200 O: 0-5 Na: 0-1

Mass	Calc. Mass	mDa	PPM	DBE	Formula	i-FIT	i-FIT Norm	Fit Conf %	C	H	O	Na
299.1620	299.1623	-0.3	-1.0	5.5	C17 H24 O3 Na	1171.9	n/a	n/a	17	24	3	1

Figure S1. LRESIMS and HRESIMS of compound **2**.

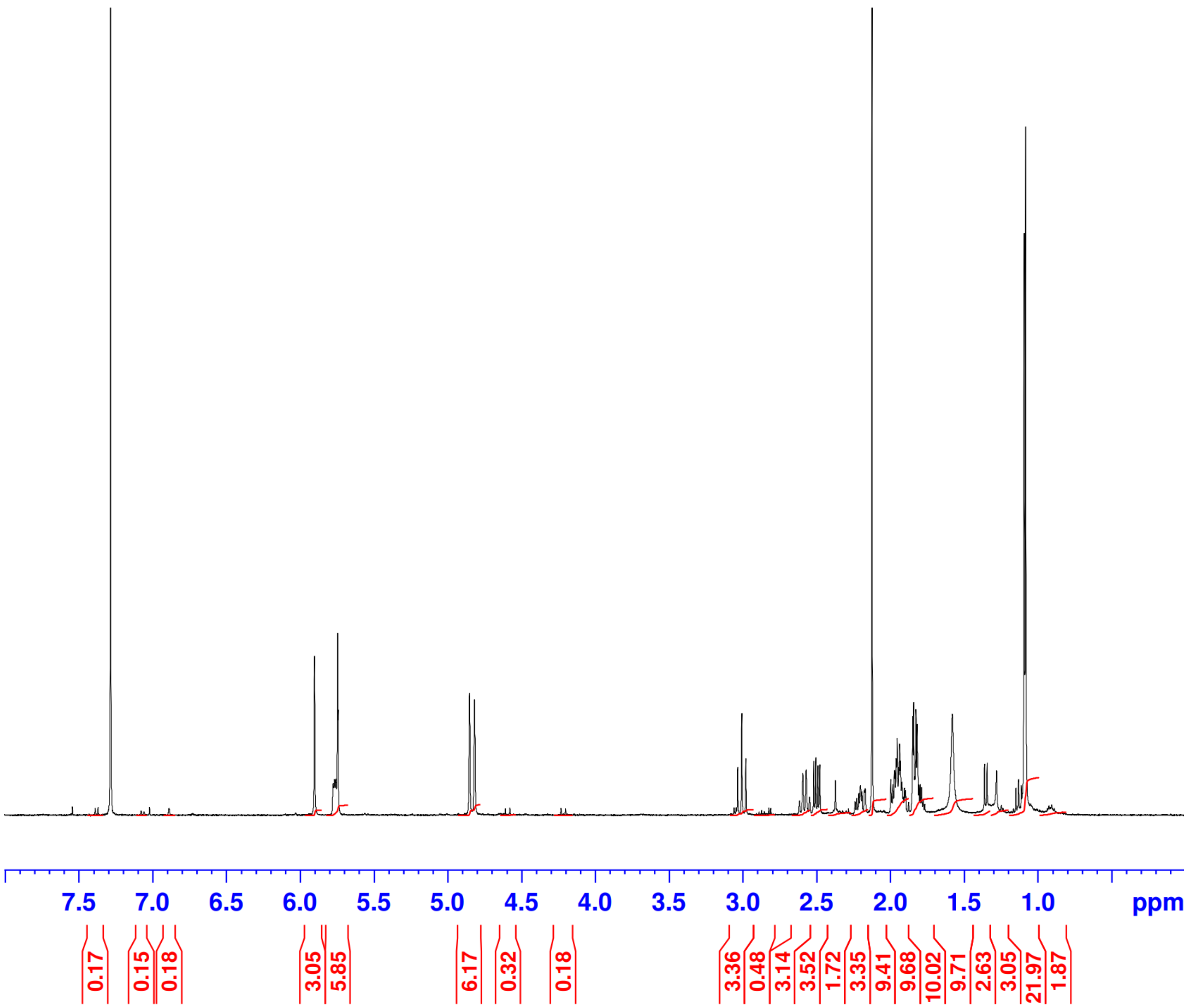

Figure S2. ^{1}H NMR spectrum of compound **2** in $CDCl_3$.

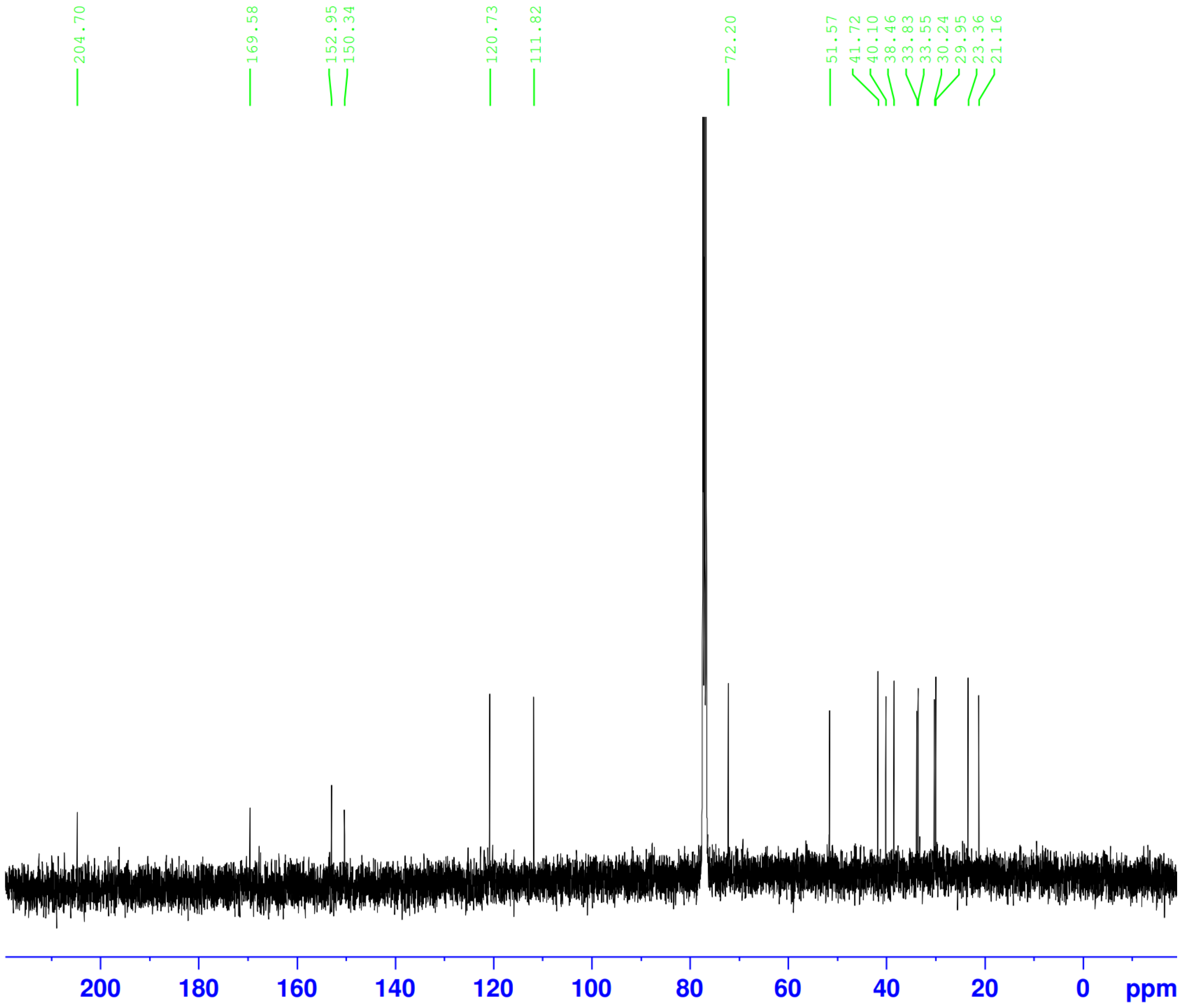

Figure S3. ^{13}C NMR spectrum of compound **2** in $CDCl_3$.

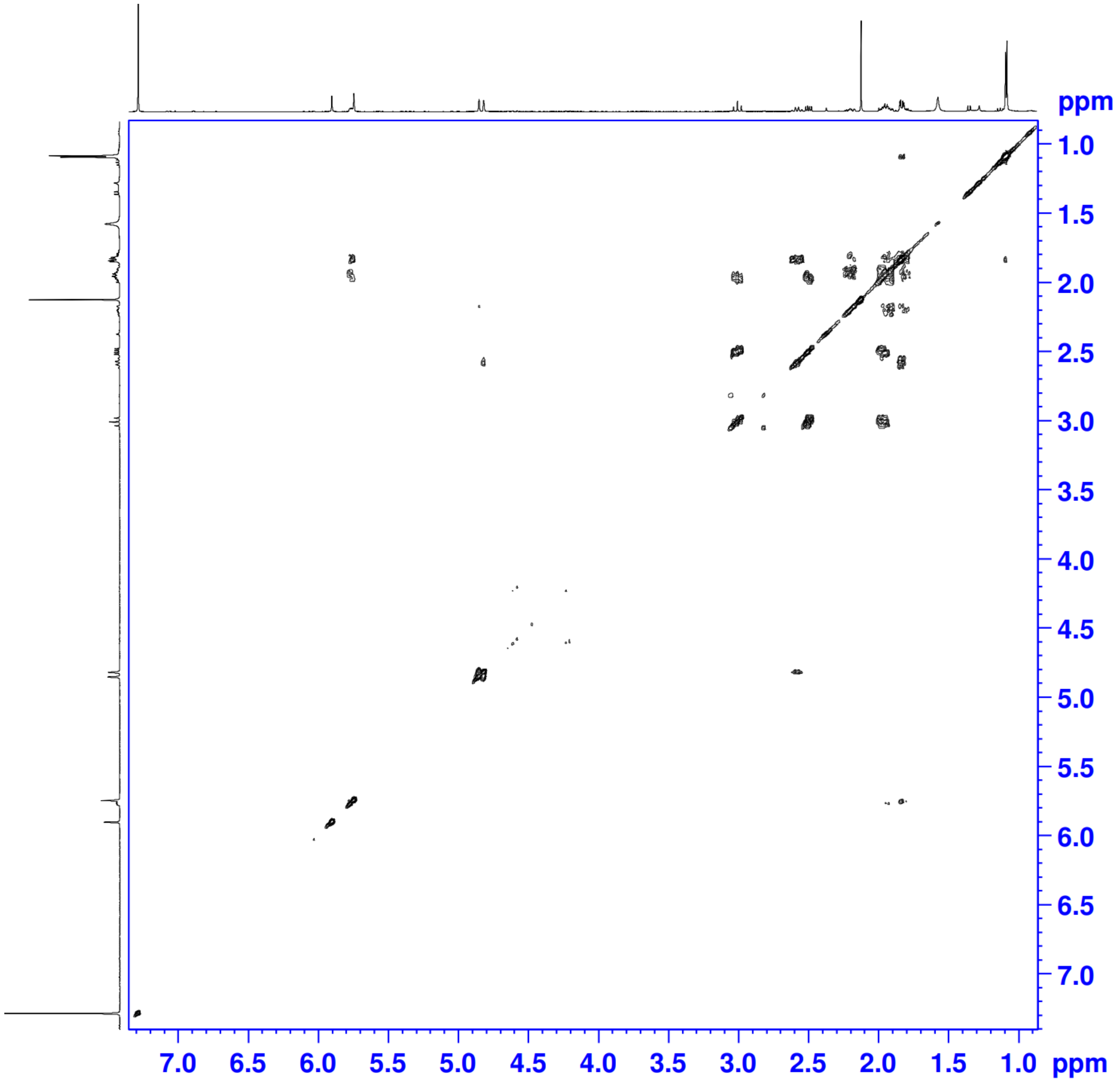

Figure S4. ^{1}H-^{1}H COSY spectrum of compound **2**.

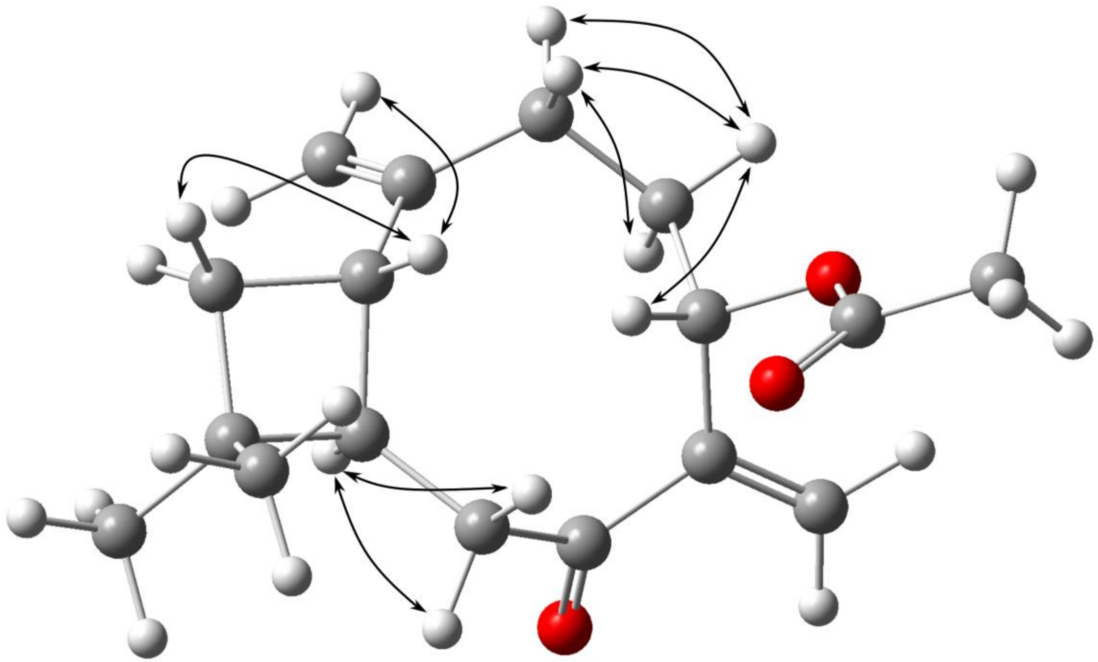

Figure S5. COSY correlations for compound **2**

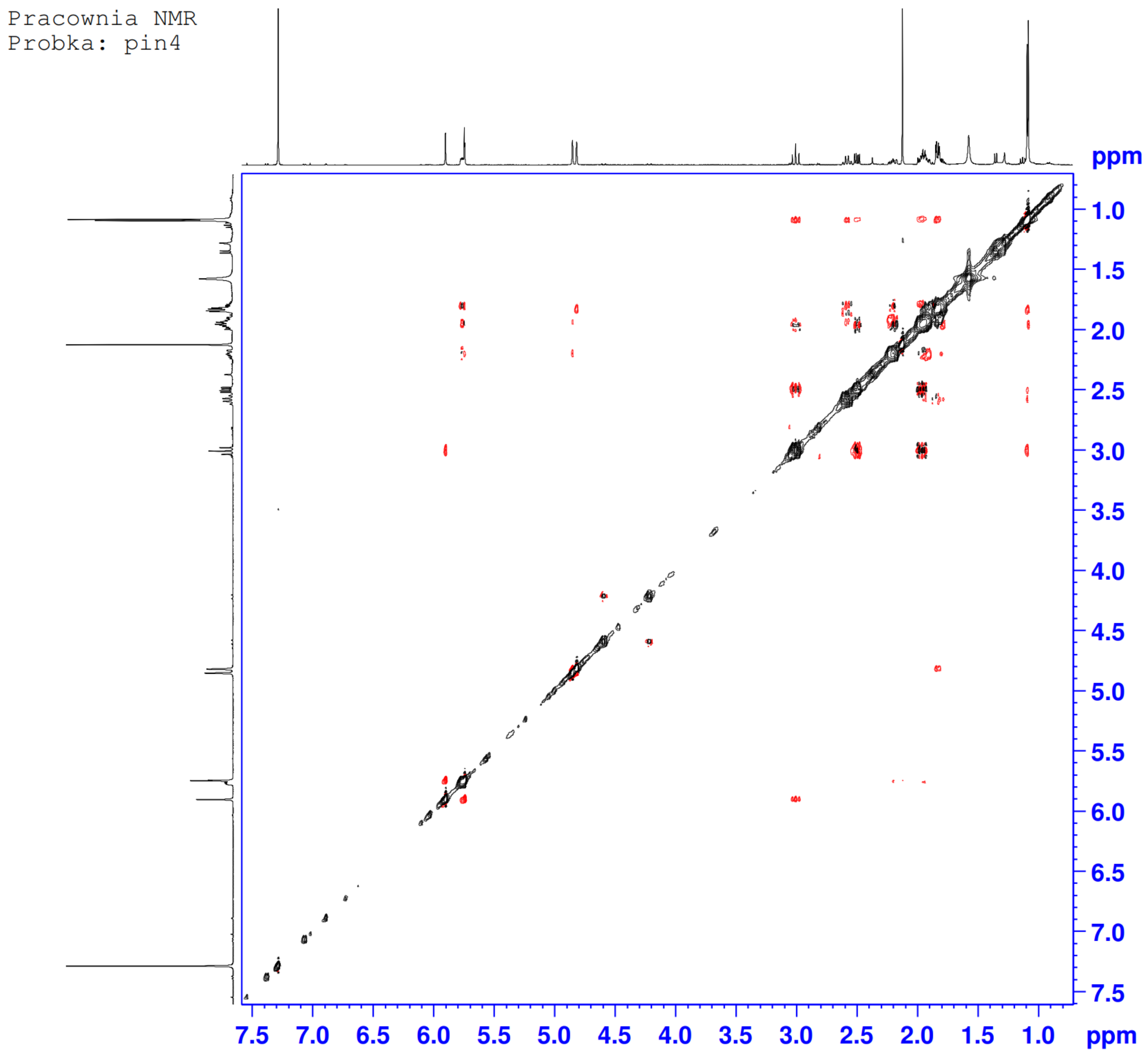

Figure S6. NOESY spectrum of compound **2**.

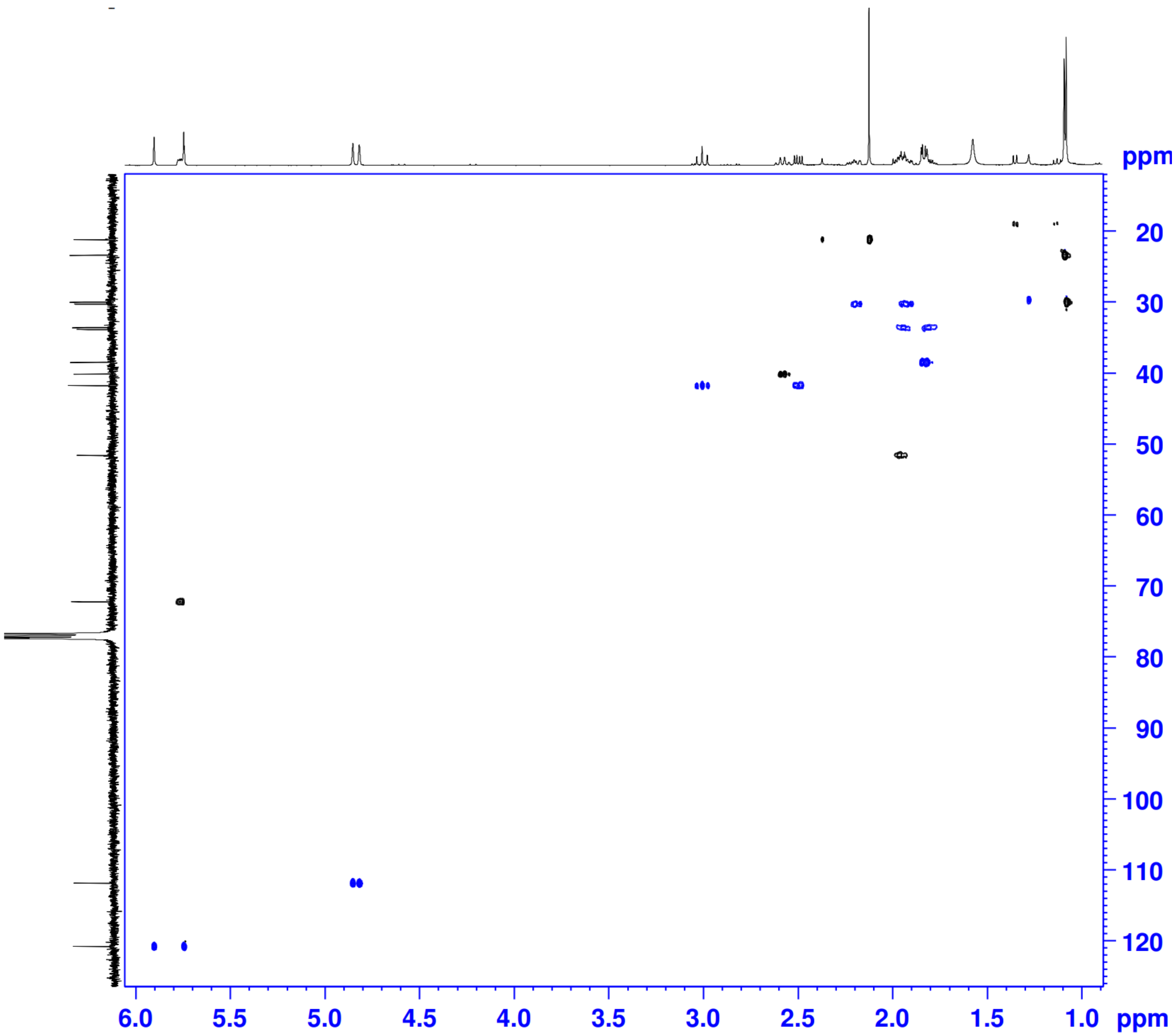

Figure S7. HSQC spectrum of compound **2**.

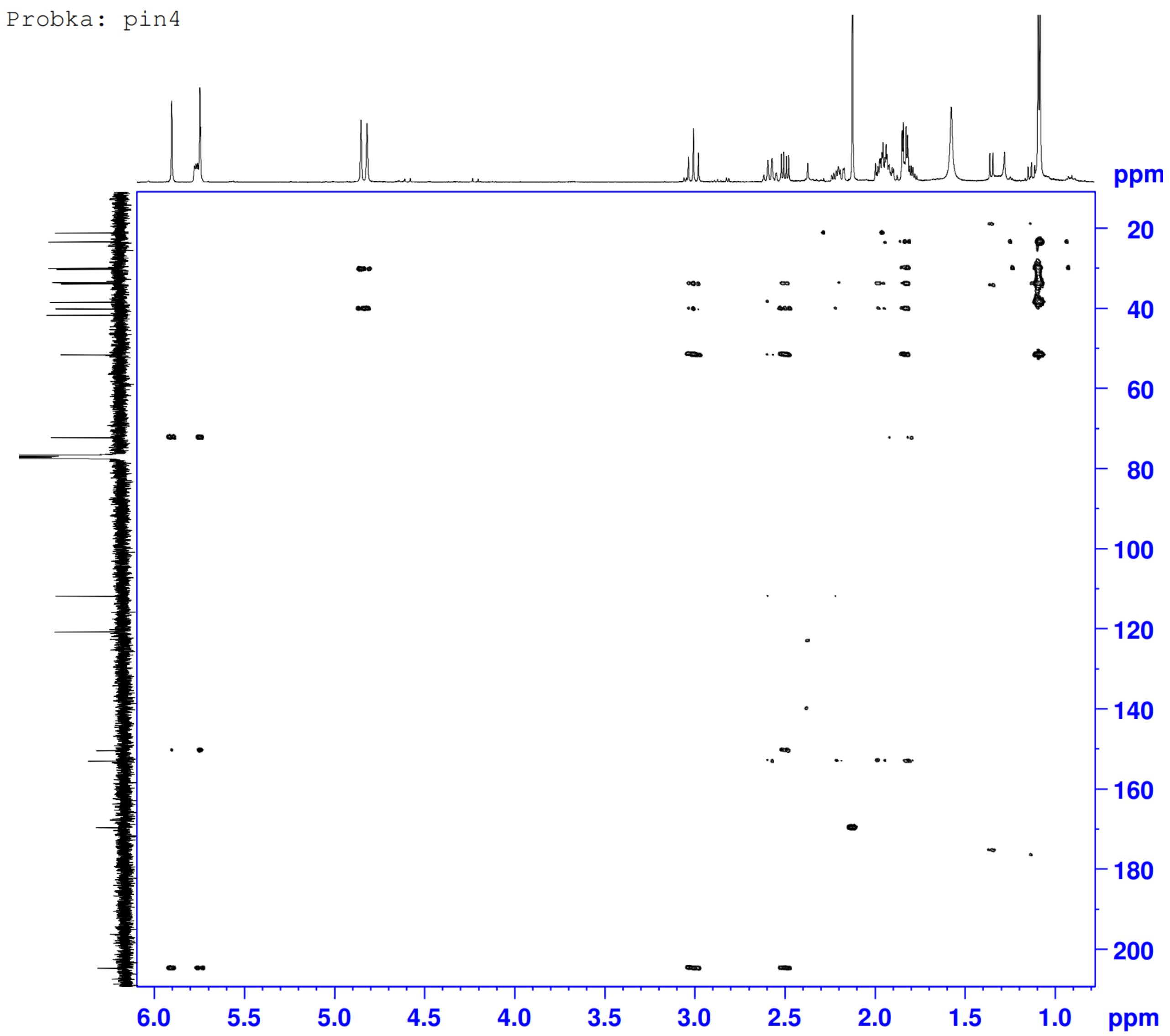

Figure S8. HMBC spectrum of compound **2**.

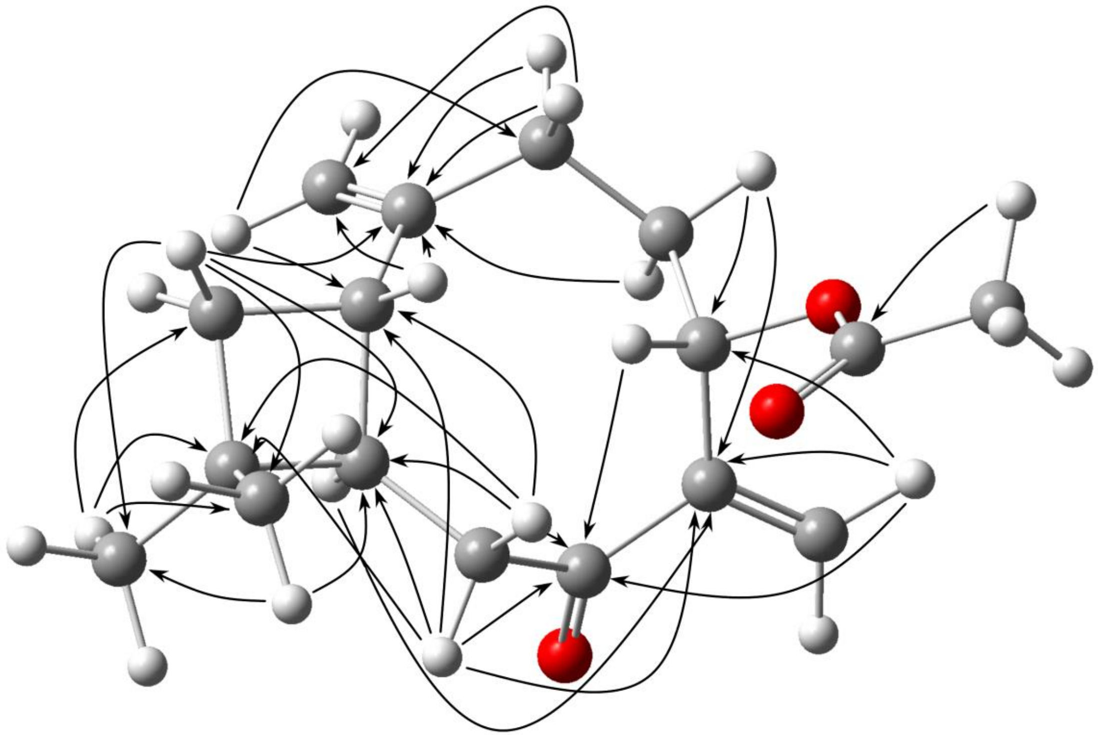

Figure S9. HMBC correlations for compound **2**

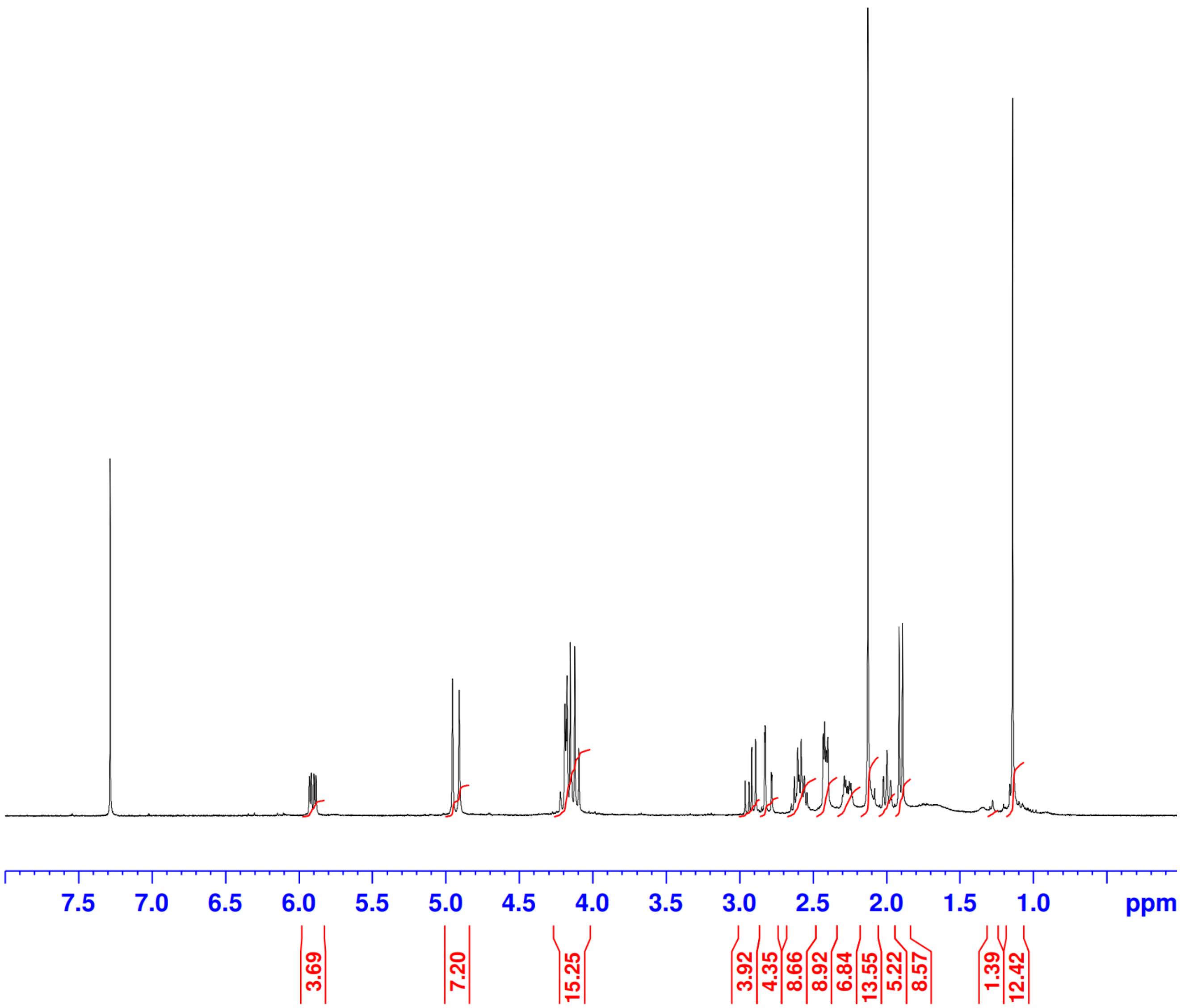

Figure S10. ^{1}H NMR spectrum of compound **9** in $CDCl_3$.

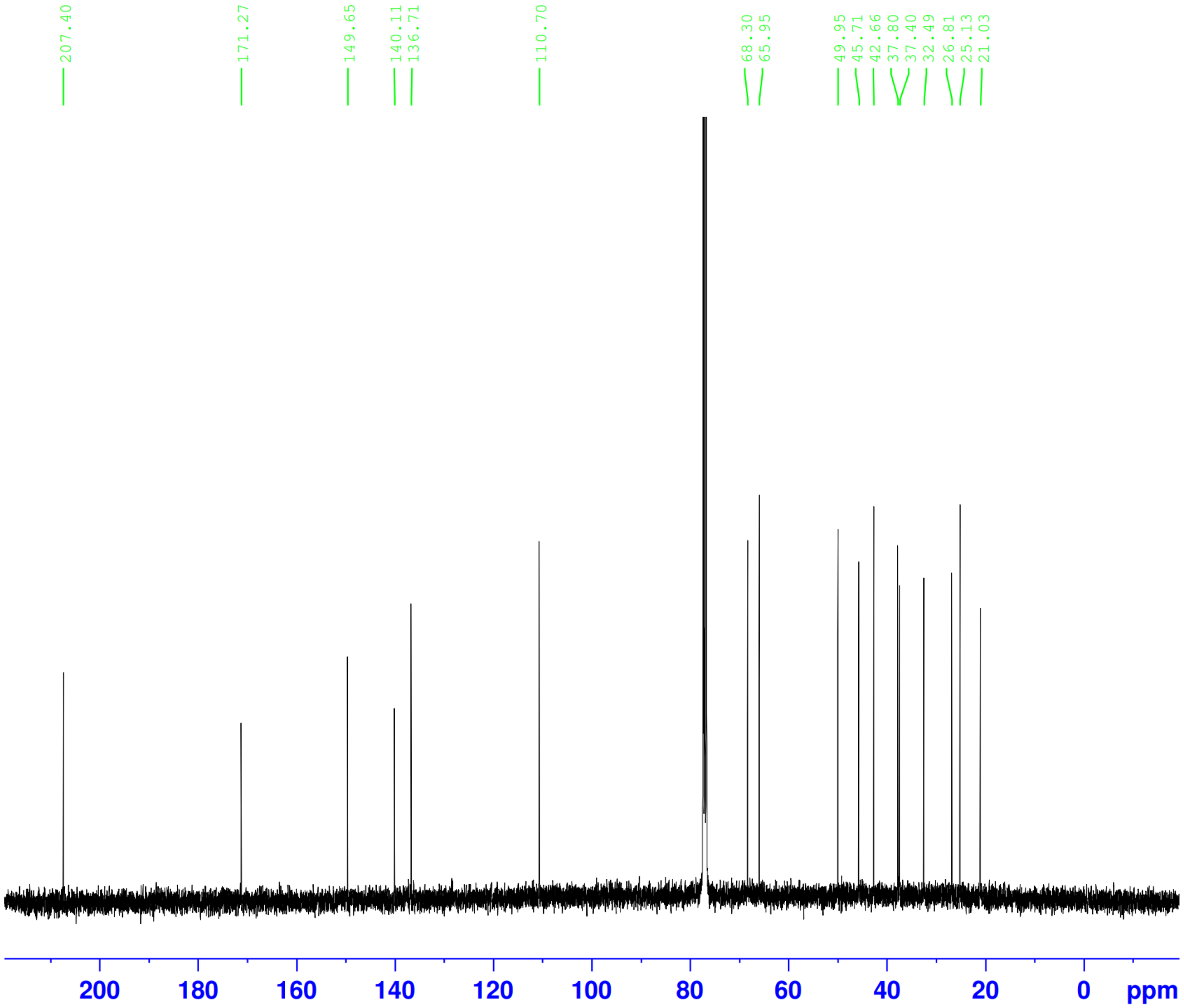

Figure S11. ^{13}C NMR spectrum of compound **9** in $CDCl_3$.

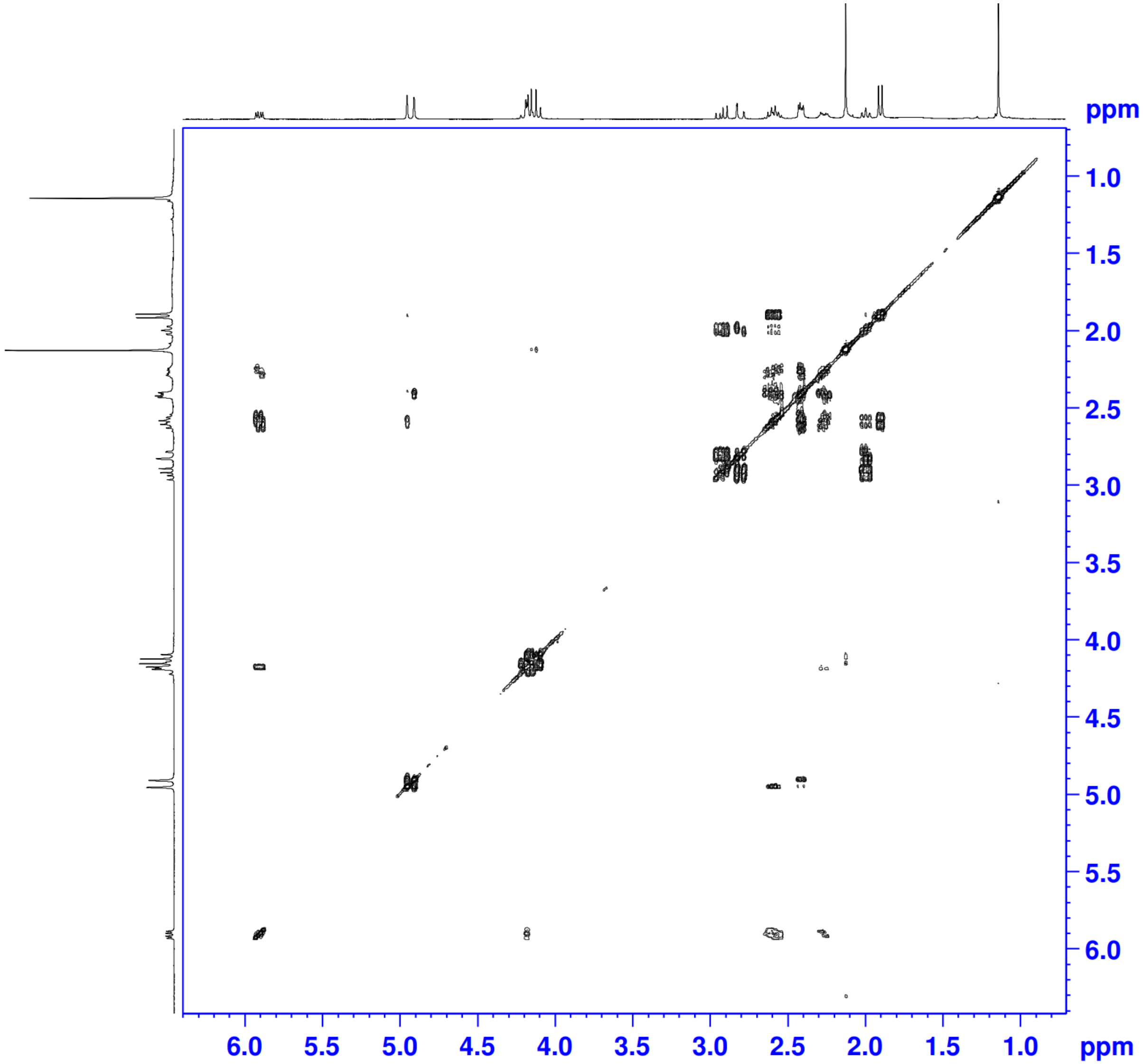

Figure S12. ^1H-^1H COSY spectrum of compound **9**.

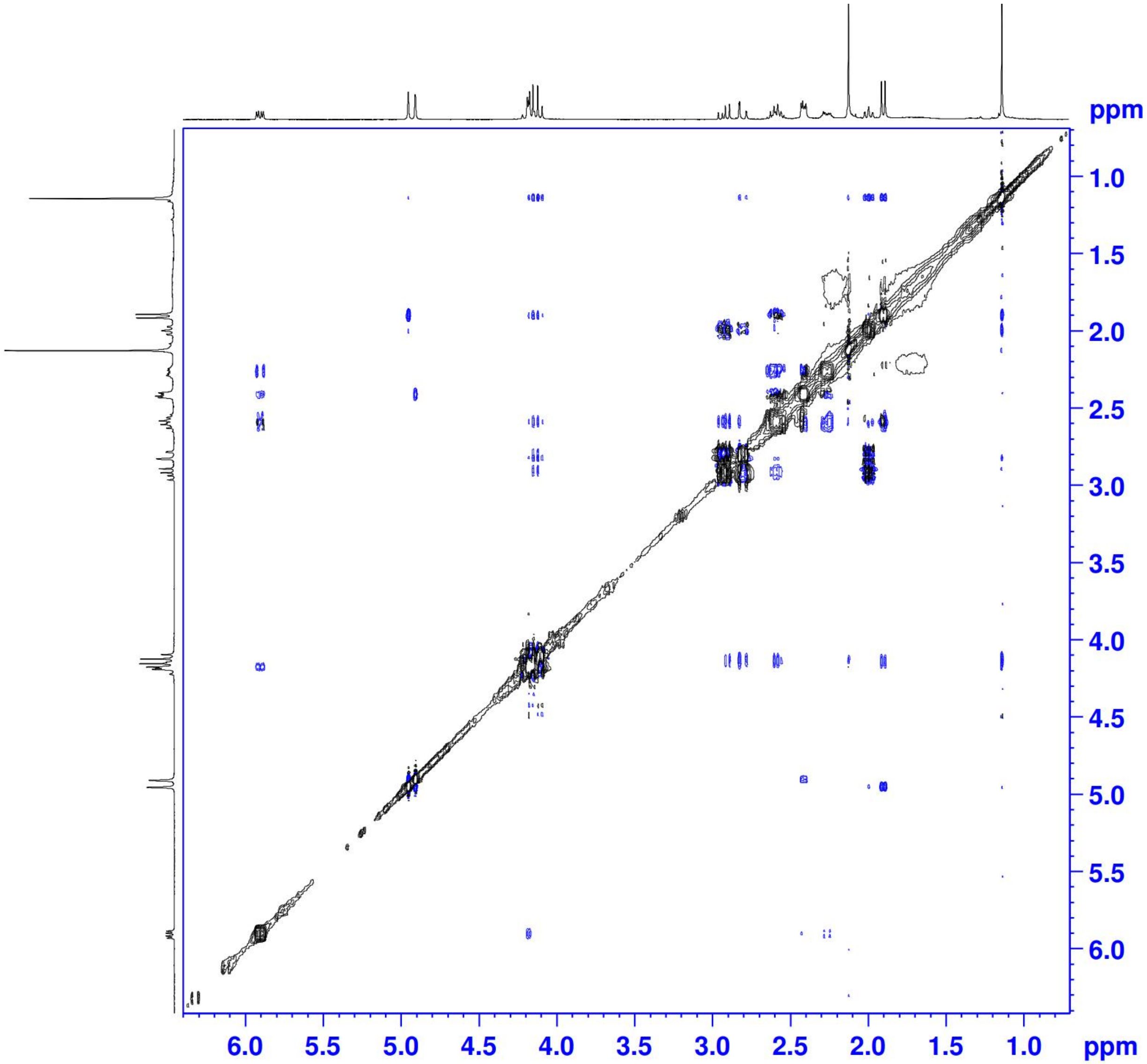

Figure S13. NOESY spectrum of compound **9**.

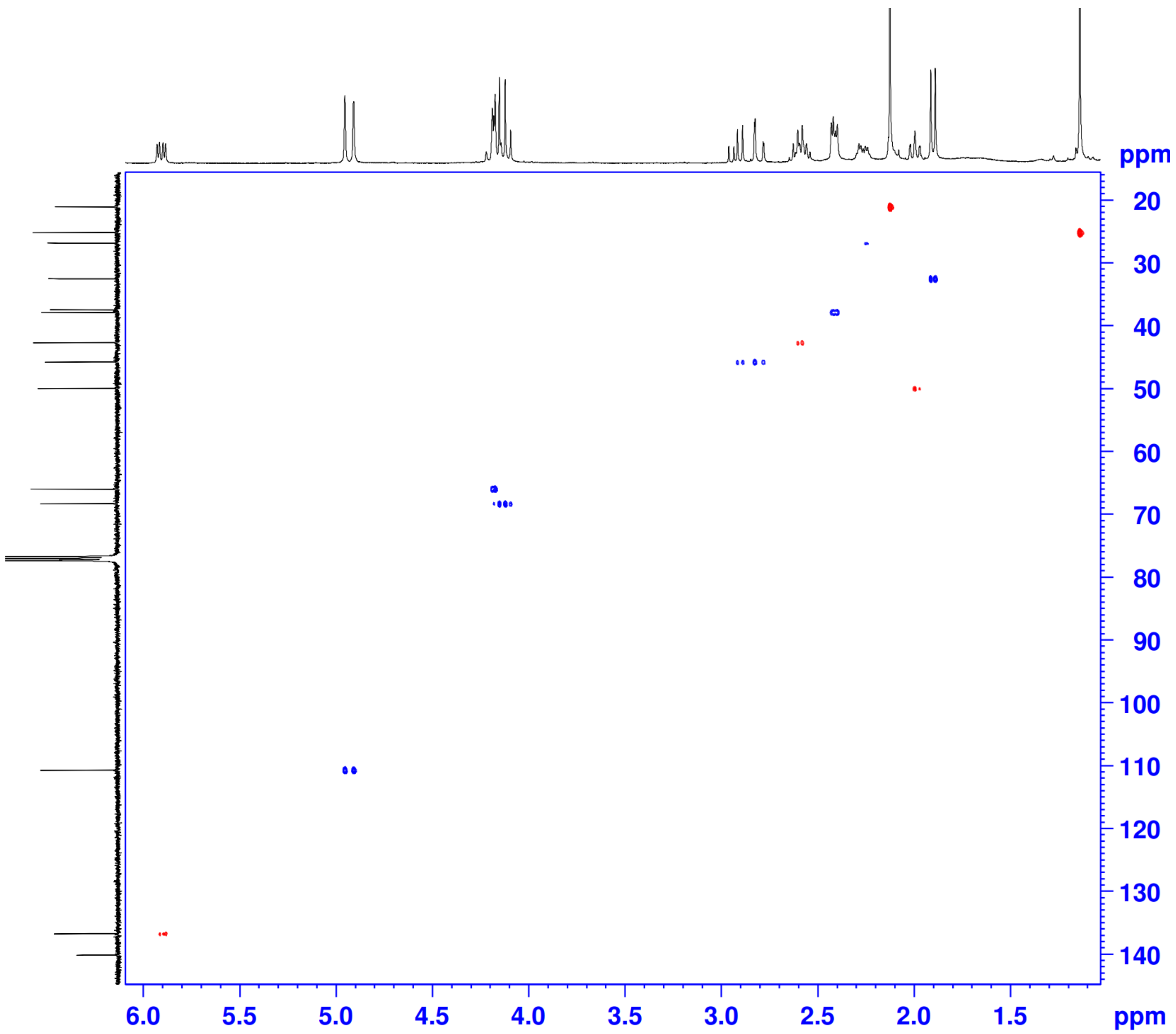

Figure S14. HSQC spectrum of compound **9**.

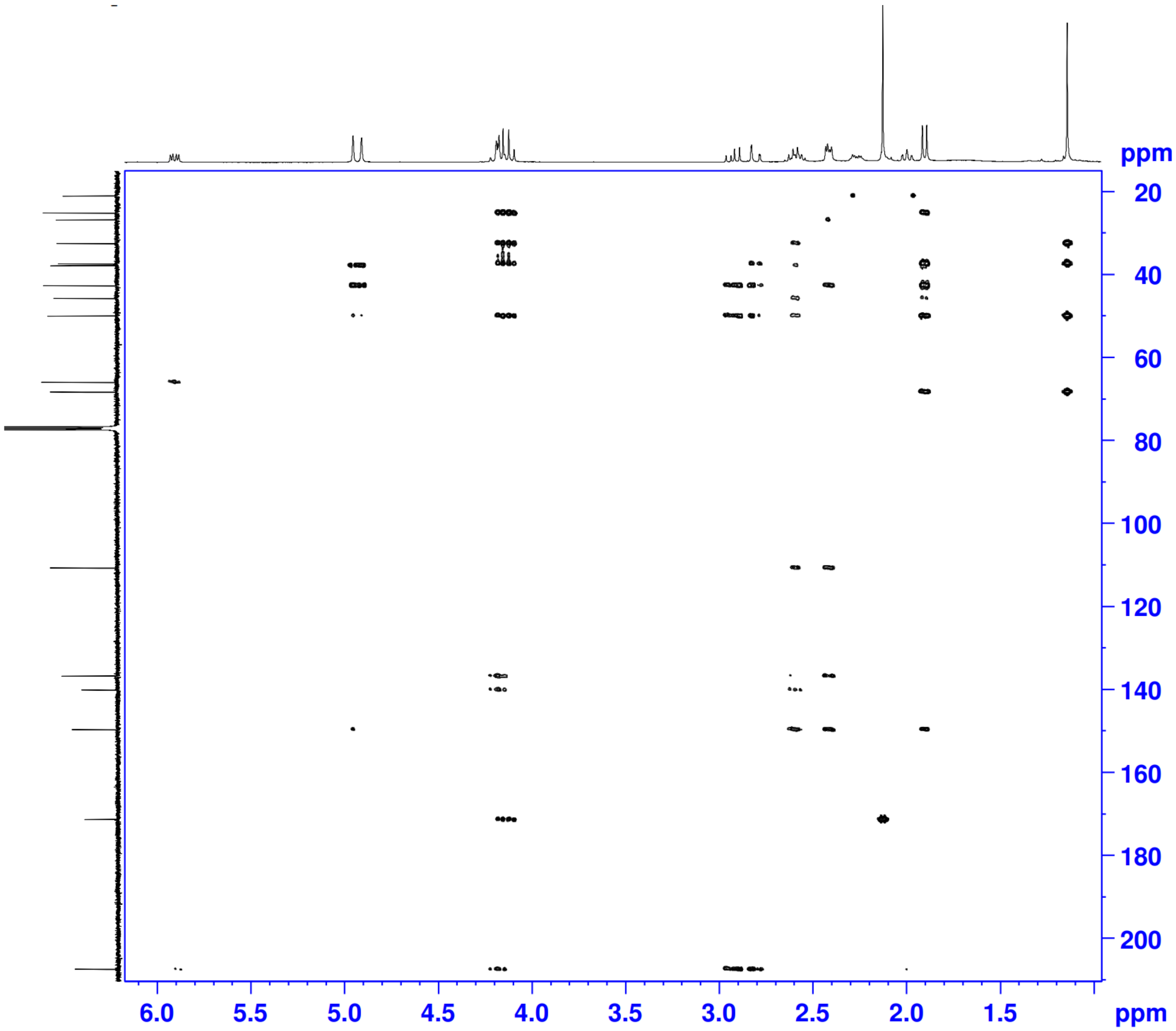

Figure S15. HMBC spectrum of compound **9**.

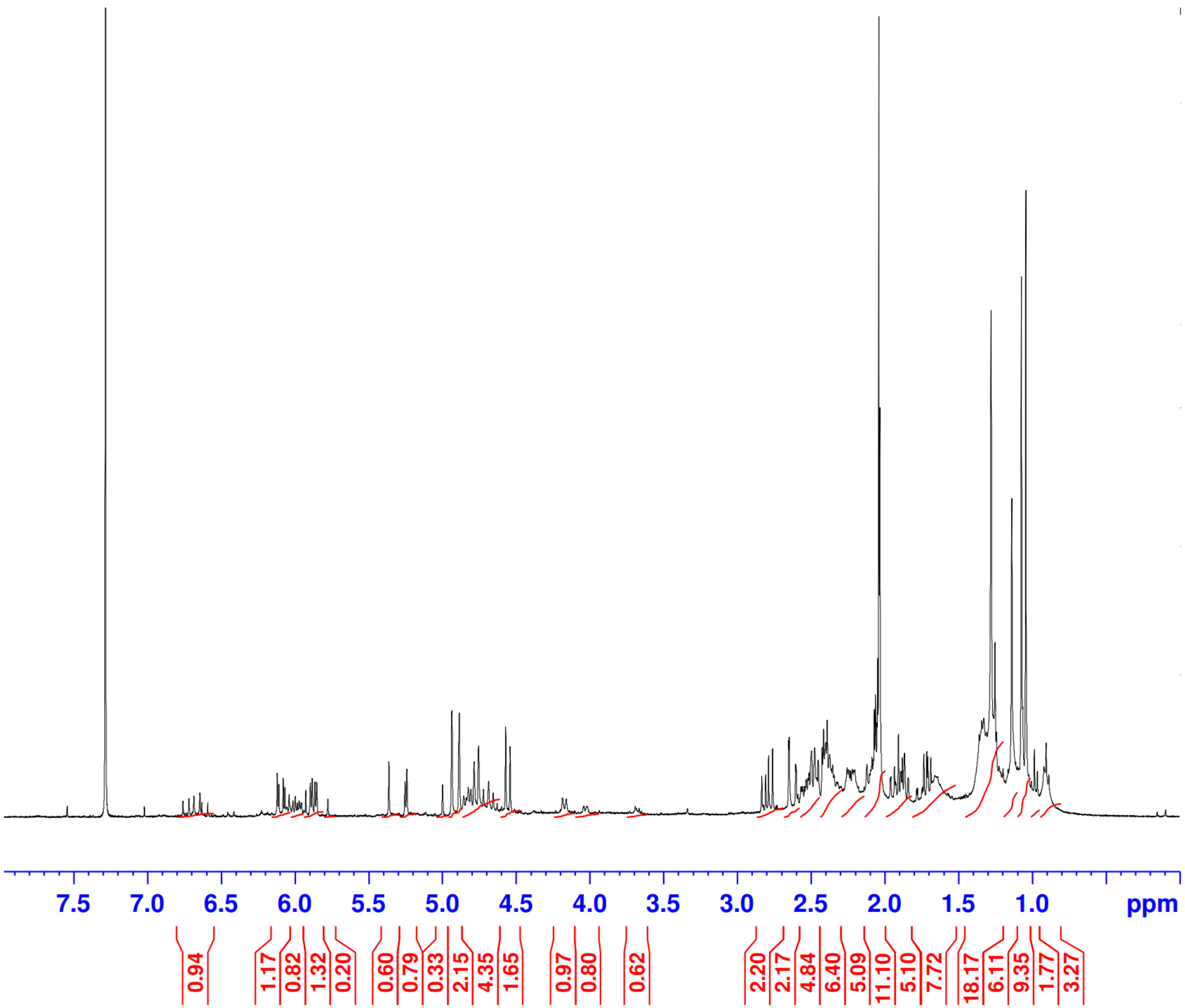

Figure S16. ^{1}H NMR spectrum of the mixture containing compound **3** in $CDCl_3$.

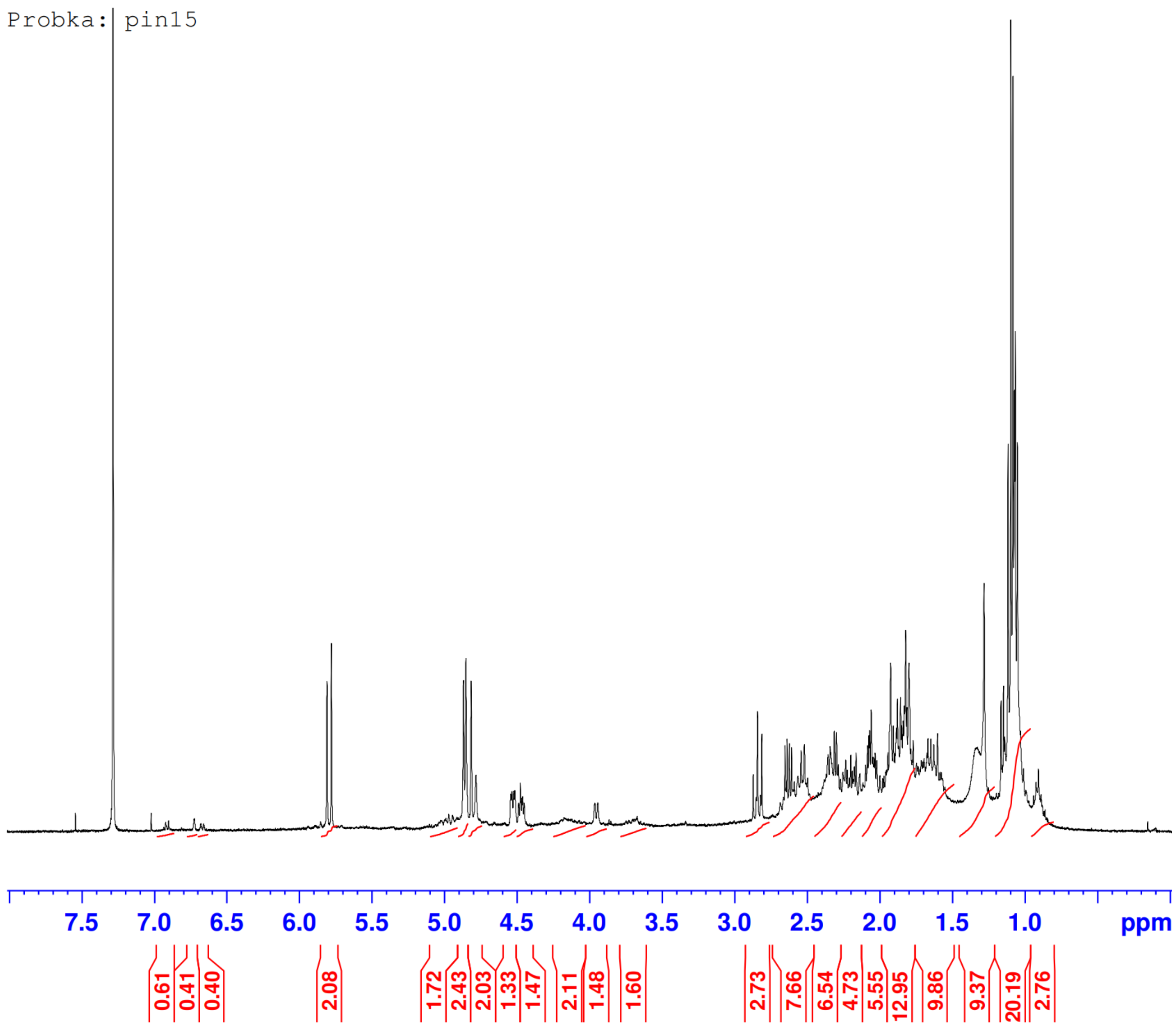

Figure S17. ^{1}H NMR spectrum of compound **7** in $CDCl_3$.

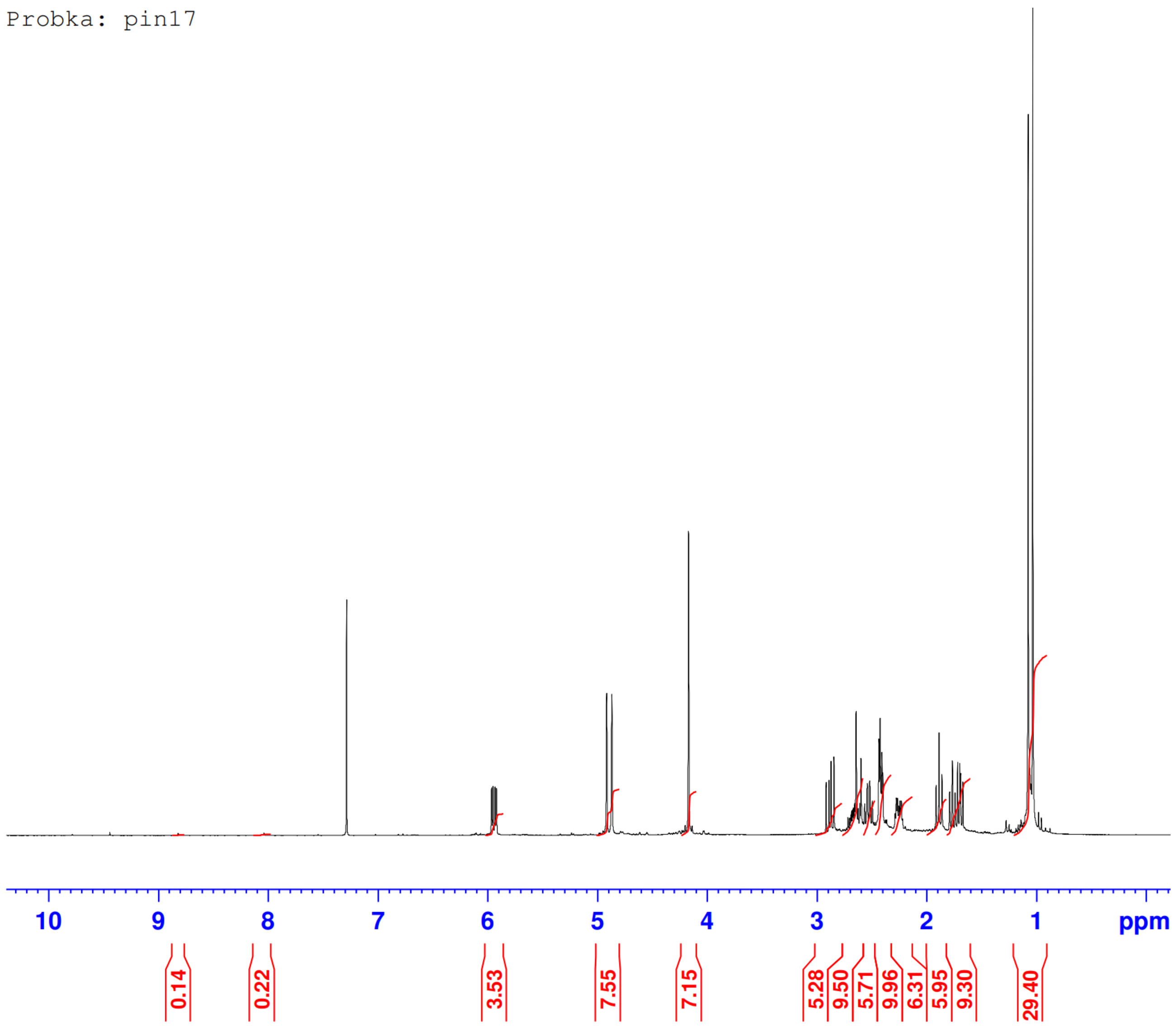

Figure S18. ^{1}H NMR spectrum of compound **8** in $CDCl_3$.

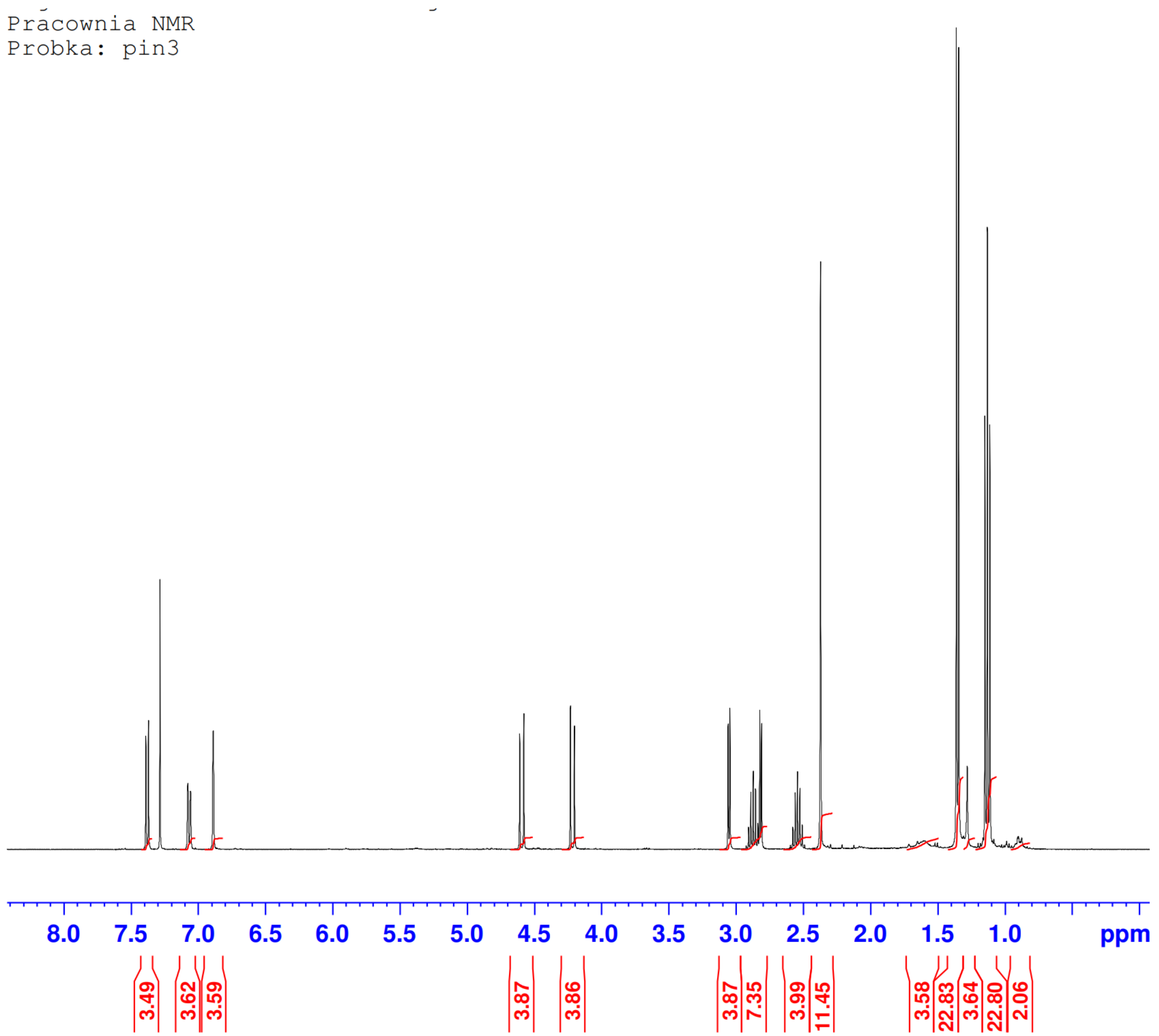

Figure S19. ^{1}H NMR spectrum of compound **1** in $CDCl_3$.

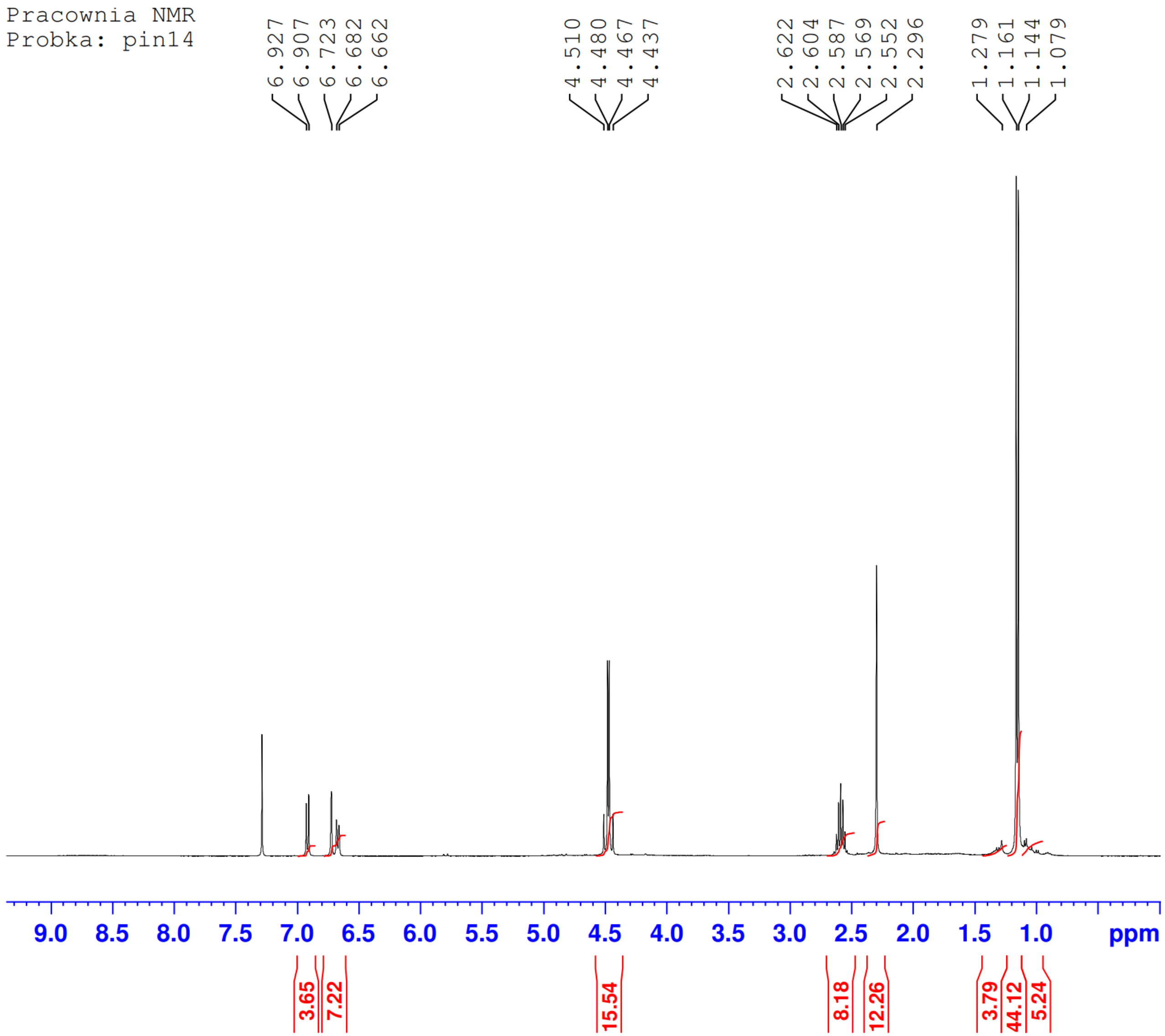

Figure S20. ^{1}H NMR spectrum of compound **4** in $CDCl_3$.

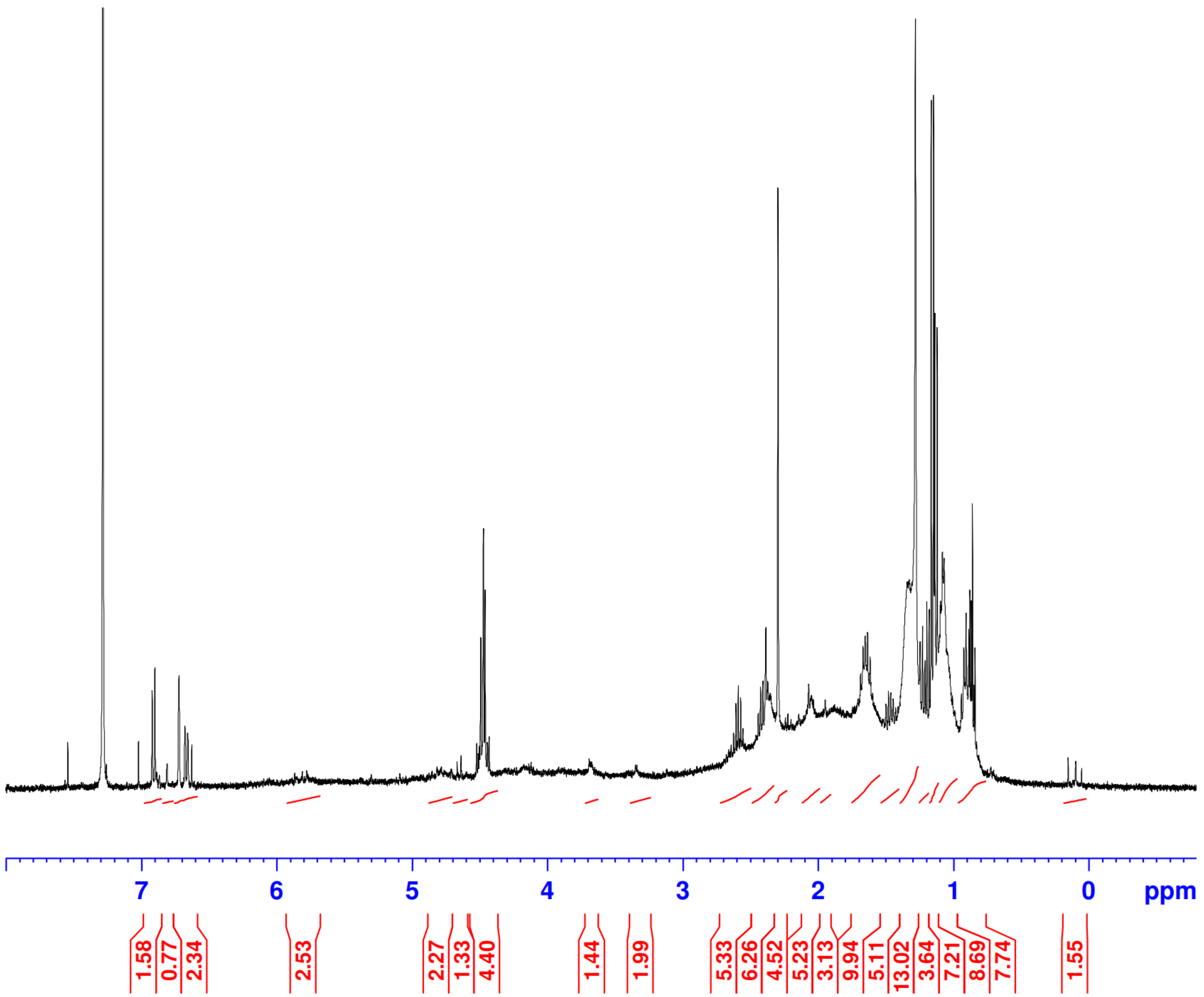

Figure S21. ^{1}H NMR spectrum of compound **5** in $CDCl_3$.

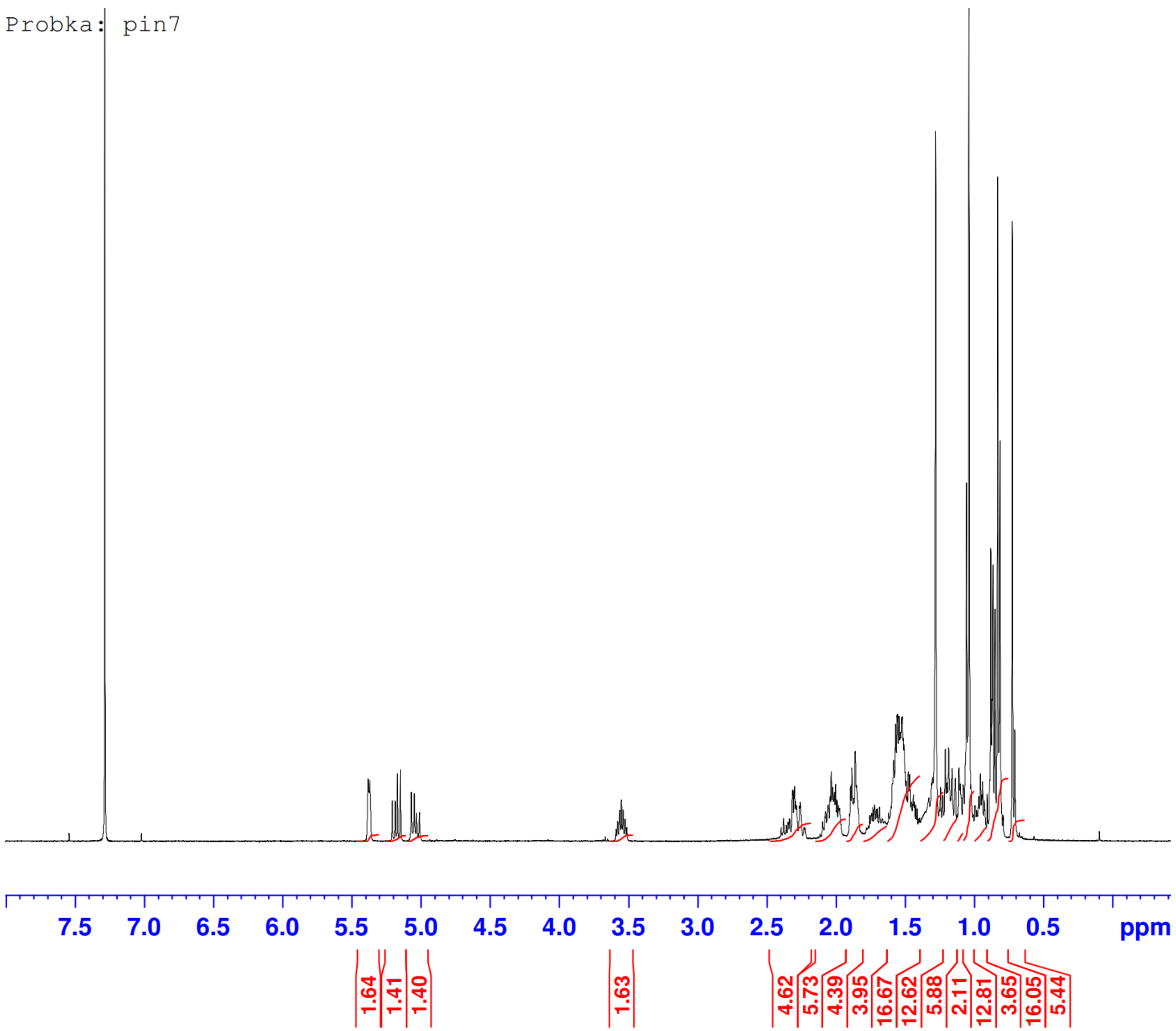

Figure S22. ^{1}H NMR spectrum of compound **6** in $CDCl_3$.

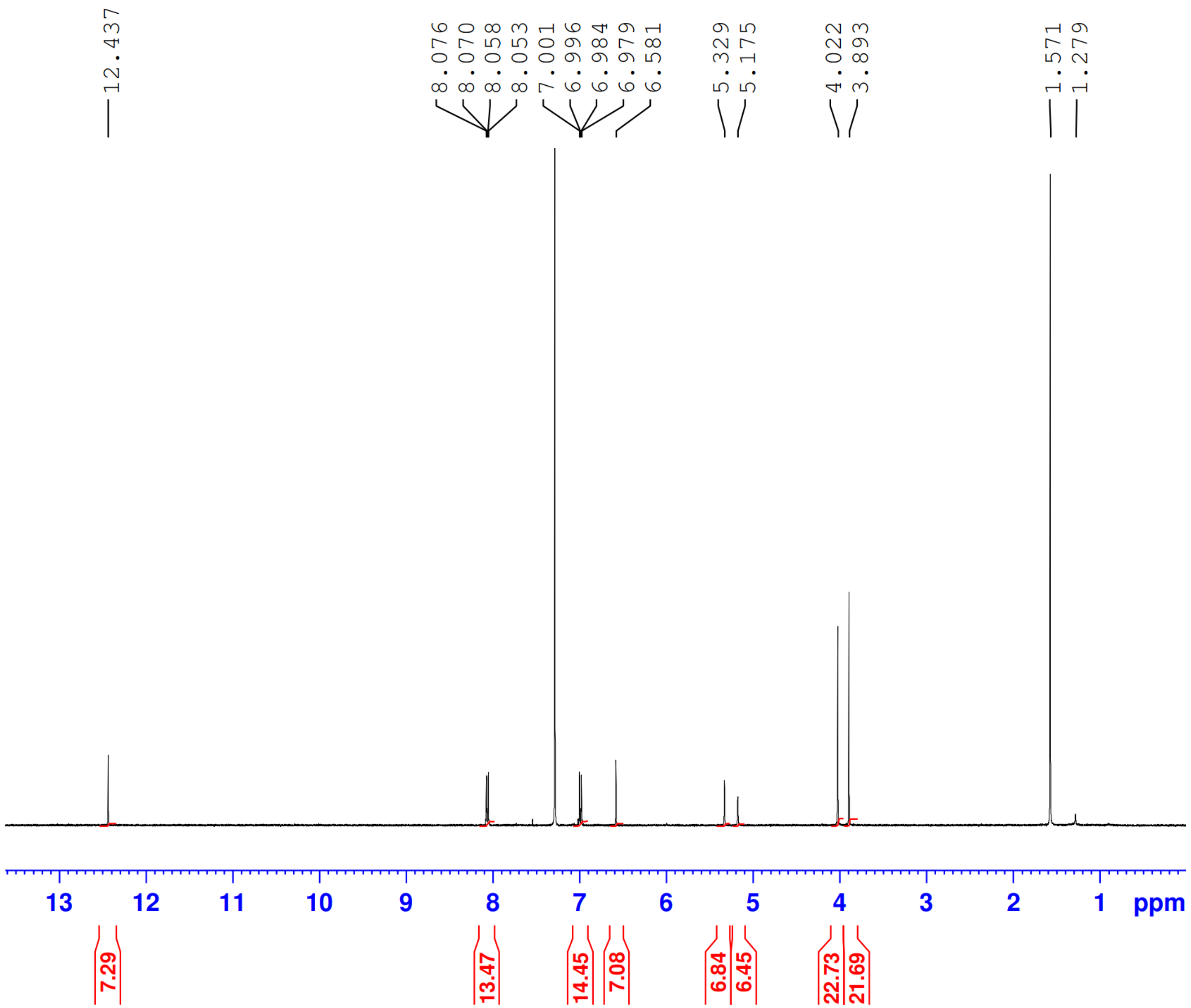

Figure S23. ^{1}H NMR spectrum of compound **10** in $CDCl_3$.

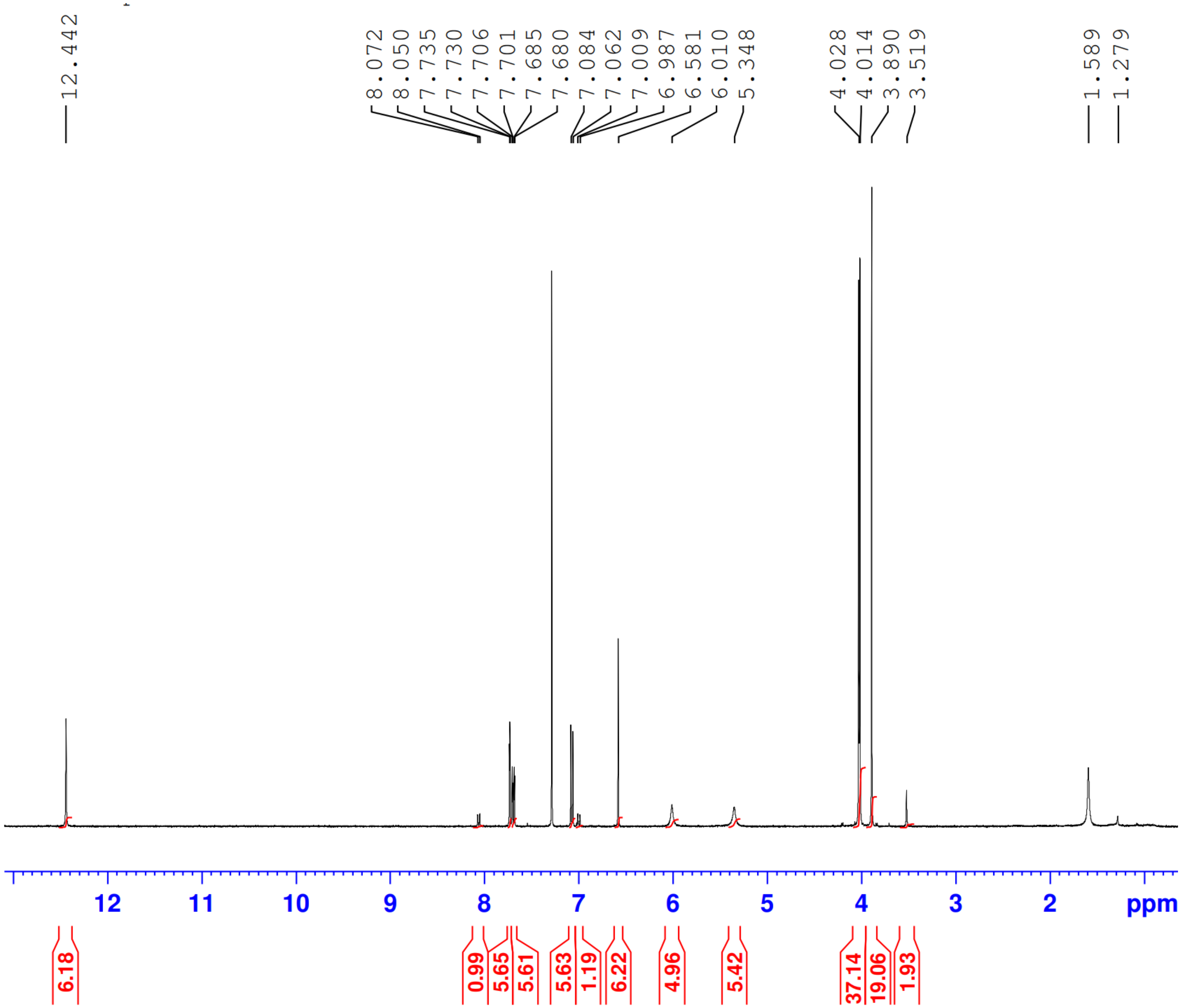

Figure S24. ^{1}H NMR spectrum of compound **11** in $CDCl_3$.

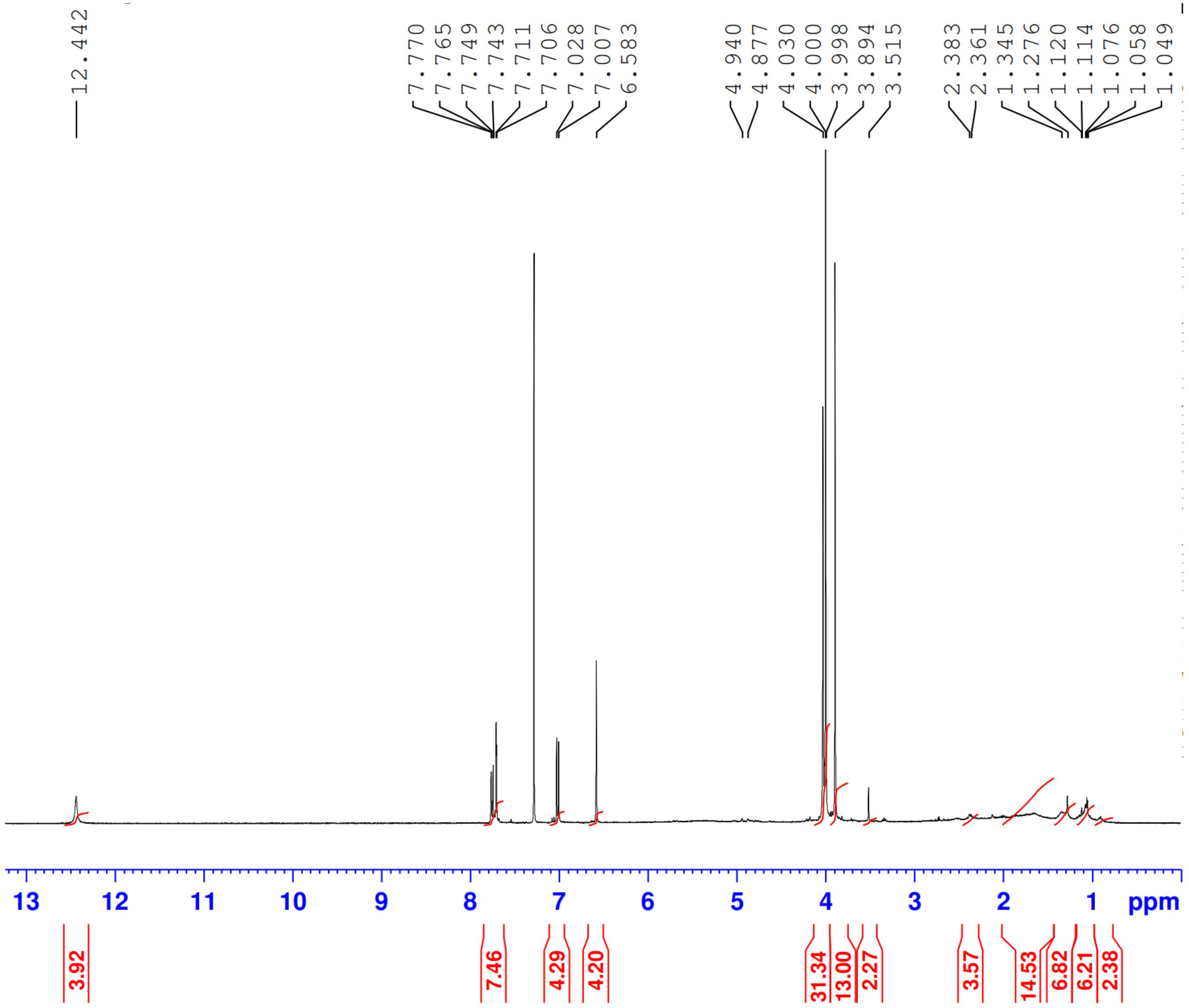

Figure S25. ^{1}H NMR spectrum of compound **12** in $CDCl_3$.

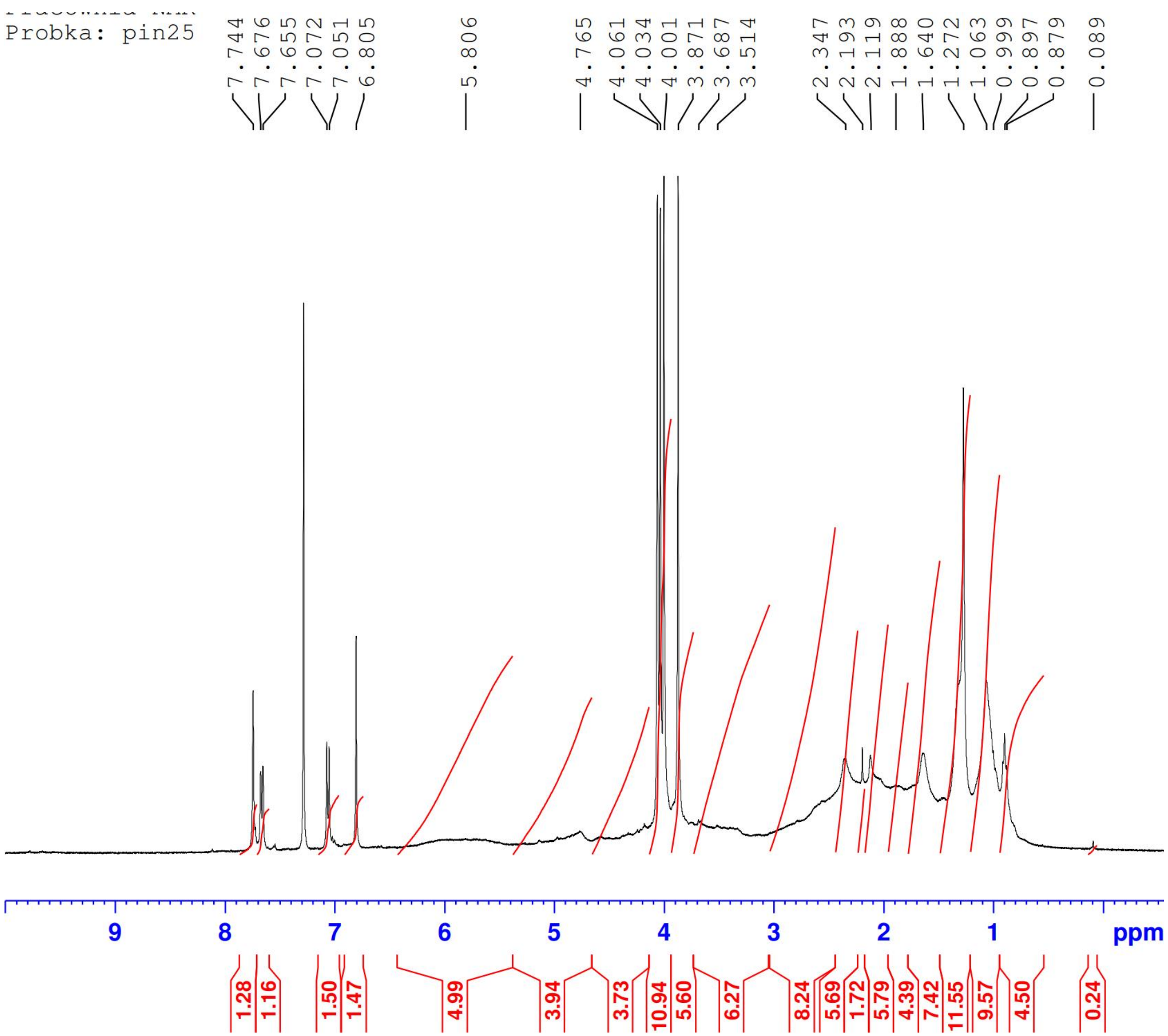

Figure S26. ^{1}H NMR spectrum of compound **13** in $CDCl_3$.

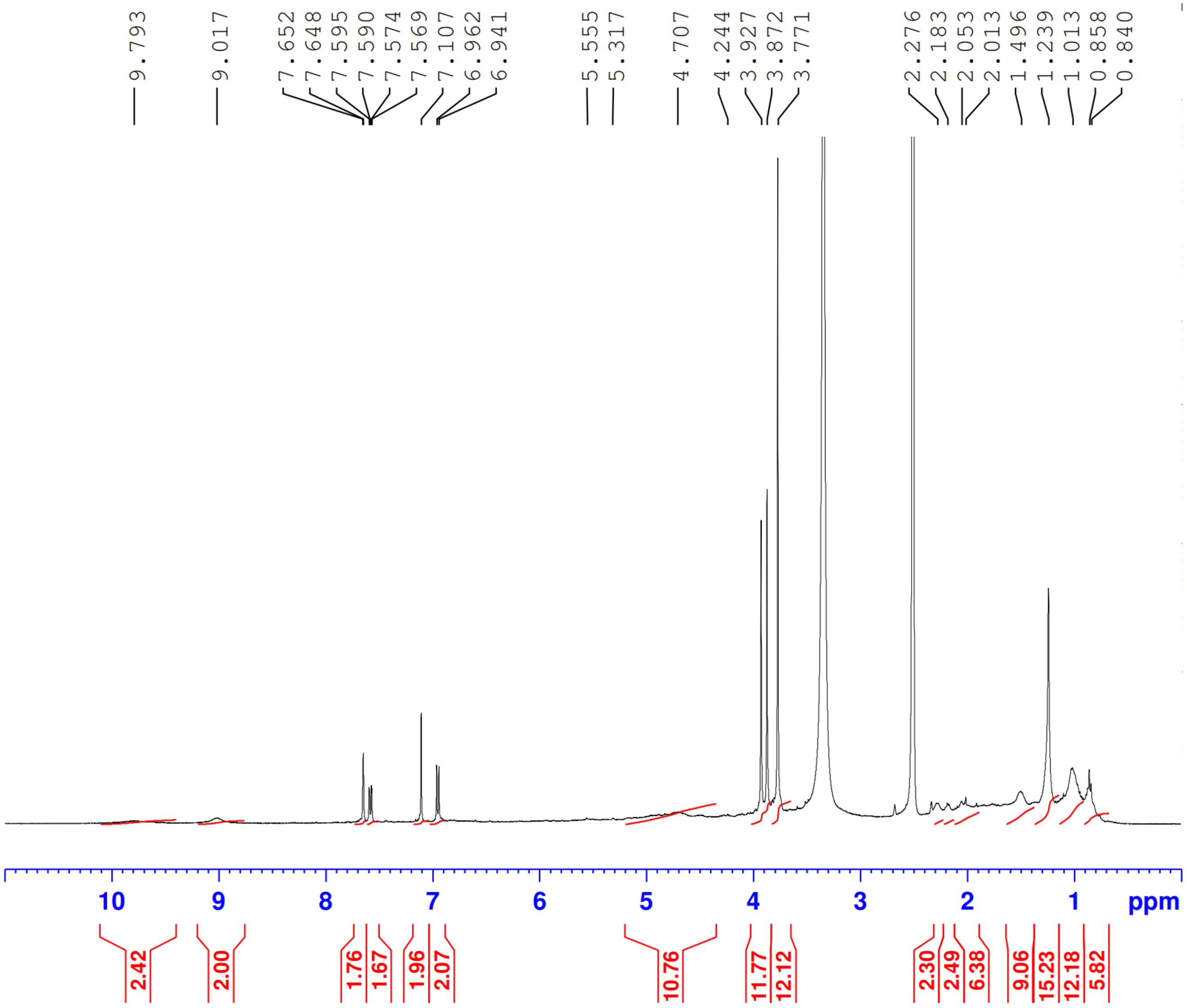

Figure S27. ^{1}H NMR spectrum of compound **13** in DMSO-d_6.
